# Effects of maternal age and offspring sex on milk yield, composition and calf growth of red deer (*Cervus elaphus*)

**DOI:** 10.1038/s41598-022-17978-3

**Published:** 2022-08-25

**Authors:** F. J. Pérez-Barbería, A. J. García, M. J. Brewer, J. Cappelli, M. P. Serrano, L. Gallego, T. Landete-Castillejos

**Affiliations:** 1 Department of Agroforestry Science and Technology and Genetics, Institute of Regional Development, Game and Livestock Resources Unit, University of Castilla-La Mancha, IREC, 02071 Albacete, Spain; 2 Biodiversity Research Institute (Oviedo University, CSIC, Principality of Asturias), Mieres Campus, 33600 Mieres, Spain; 3 Biomathematics and Statistics Scotland (BioSS), Craigiebuckler, Aberdeen, AB15 8QH Scotland, UK; 4 Departamento de Producción Agraria, Escuela Técnica Superior de Ingeniería Agronómica, Alimentaria y de Biosistemas, Polytechnic University of Madrid, 28054 Madrid, Spain

**Keywords:** Ecology, Zoology

## Abstract

Differential maternal allocation theory states that mothers will invest more heavily in the offspring sex that will secure higher reproductive output. Senescence theory is concerned with the gradual deterioration of physiological function with age. We analysed the offspring sex-dependent response of calf growth and milk traits to mother age in an Iberian population of captive red deer *(Cervus elaphus)* using a 22 year time series longitudinal data set. Previous studies revealed that there was little evidence for the differential allocation theory on milk traits and that most studies lacked proper control for confounding factors. Our results indicated that (i) calf growth was offspring male-biased, negatively affected by mother age and positively influenced by mother weight and parity, and (ii) there was no support for differential allocation offspring sex-dependence in milk traits (yield, energy density, fat, protein and lactose content). Our findings suggest that maternal allocation responds to offspring energy requirements, which are mainly driven by offspring body weight, and contingent on mother age and weight and previous maternal reproductive effort.

## Introduction

The behavioural and physiological activities associated with reproduction generally evolve towards improving the fitness of the offspring at a cost to the parent^1^. Evans^2^ collectively termed all these activities "parental input", and he defined it as a direct measure of the care or resources provided to the offspring, regardless of the cost to the parent. Parental input differs from parental investment as defined by Trivers^1^, for this author investment in an offspring must increase the probability that the offspring will survive always at the expense of the parents' ability to invest in other offspring.

In vertebrate species in which sexual dimorphism in body size is partially achieved during the period of parental care, differences in resource allocations to offspring by parents are expected, but there are also evolutionary aspects, related to future reproductive offspring output, by which parents may vary the allocation of resources to offspring of different sex^3^. The differential maternal allocation theory^4,5^ states that mothers in better body condition will invest in offspring of the most costly sex and higher reproductive output, while mothers in poor condition will invest in offspring of the sex for which condition is less dependent on mating success. This is based on the following premises^3,5^, (i) maternal body condition has a positive effect on offspring growth, the latter being instrumental in reproductive fitness^6^, (ii) variance in breeding success in polygynous species is greater in males than in females^7^, and (iii) adult size, which is determined by early growth and retained into adulthood, has a greater effect upon male than upon female offspring reproductive success^3^.

Polygynous males´ mating success greatly depends on their competitive fighting skills against other rival males to capitalise large harems^8^; body size is a good predictor of success in fighting^9^ and the key period in postnatal growth takes place during lactation^10^. Contrastingly, a female´s reproductive output is mainly determined by the length of their reproductive lifespan^11^, as the number of offspring produced in a reproductive season is small, in many polygynous deer species limited to one offspring^12^.

Senescence, the process of deterioration of physiological function in old age^13^, has been assumed as responsible for observed declines in survival and reproductive performance in animal populations^13,14^. Rates of senescence are not necessary age-related but depend on previous experiences, life history, and rates of accumulation of physiological damage, and may be better predicted by years to death rather than age^11^.

Milk provides the neonate with water, minerals, nutrients and antibodies, which enable the neonate to pass from an extremely mother-dependent condition to a self-reliant nutritional state, and it is key for the development of a mother–offspring bond^15^. Lactation is the greatest energetic expenditure of reproduction for mammalian females^15^ and the maternal ability to sustain the costs of lactation are not just those related to diet and intake^16^ but those influenced by mother and offspring life history factors, especially those acting on condition and energy requirements^17^.

Most studies on parental resource allocation have focused on (i) the offspring sex-ratio^18^, (ii) offspring growth^19^, and (iii) maternal care^3^, but despite the importance of milk provisioning as a key maternal reproductive life history trait, little is known about changes in resource input related to maternal age and allocation in milk traits to offspring of different sex. Furthermore, amounts of investment have usually been estimated from measures of parental input and this has led to a blurring of the distinction between the two concepts^1,2^. It has been claimed that in some species of primates and ruminants milk yield and milk composition is offspring sex-dependent, but the evidence is not consistent across studies^20–25^.

We use 22 year longitudinal data of a population of 156 captive red deer hinds and 635 calves to assess reproductive input related to maternal age in milk yield and milk composition (1715 milking records) and calf growth (10,297 body weights), controlling for a number of effects that have not been properly analysed in previous studies. We based some of our hypotheses and predictions on the premises that variation in milk composition is driven by trade-offs between environmental biotic factors (food quality and availability), physiological constraints on milk synthesis, and selective forces to maximize offspring fitness^5,26^. We hypothesise that milk yield, milk composition and calf growth are related to mother age (H1) contingent upon maternal allocation history (H2), mother´s condition (H3), offspring sex (H4), reproductive phenology (H5) and that the plasticity of milk composition cannot respond to individual-specific reaction norms, such as differential maternal allocation, because of environmental trophic constraints to which captive deer are exposed (see predictions in Table 1 and Material 1 in Supplementary Information 1). We compare our findings with data from previous studies and argue that differences between studies are due to lack of control of some key factors.

**Table 1:** Hypotheses on the effect of maternal age (H1), maternal allocation history (parity) (H2), maternal condition (H3) and differential input allocation (H4-5) and parturition date (H6) and predictions (P1–P6) on maternal reproductive life history traits (milk yield, milk energy density, milk composition and offspring growth) in captive Iberian red deer.

	Milk yield	Milk energy density	Milk composition	Offspring growth	
H1. Mother age	H1	H1	H1	H1	P1. Improvement through early life, plateau in prime age, and then suddenly decline
H2. Parity	H2	H2	H2	H2	P2. Improvement through early life, plateau in prime age, followed by a maintained gradual decline that is favoured because of reproductive experience, and then a sudden decline
H3. Mother condition	H3	H3	H3	H3	P3. A general increase with maternal condition
H4. Offspring sex	H4	H4	H4	H5	P4. Milk energy and composition will not differ between offspring sexes, as in captive deer there is very little choice for mothers to select diets that enable them to modify milk composition P5. Mothers will maximise offspring growth of both sexes, consequently, offspring growth will be greater in males and in females of dimorphic species in body mass
H5. Parturition date	H6	H6	H6	H6	P6. Early parturition dates generally correspond with mothers in good condition but also with synchronising lactation with vegetation growth, in well fed captive deer the latter effect might have little effect on maternal reproductive input

## Results

### Calf growth

**Figure 1:**
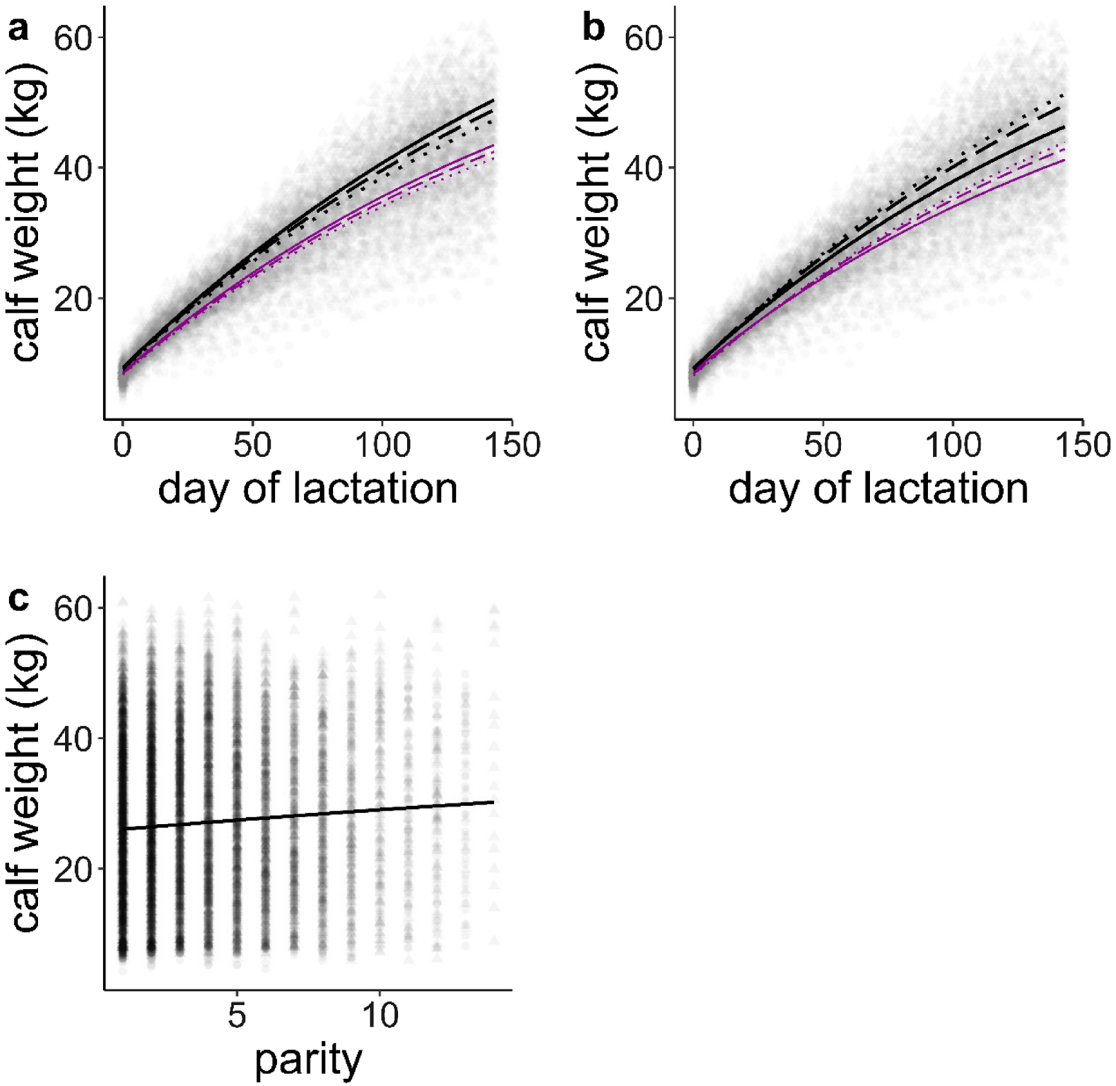
Prediction of male and female red deer calf weight against day of lactation contingent upon hind weight (**a**), hind age (**b**) and parity (**c**). Predictions are based on the model in Supplementary Information 3 Table 1 after fixing the main effects at their mean values and hind weight and hind age at their quartiles Q1, Q2 and Q3. (**a**) thin-magenta line: female calf; thick-black line: male calf; dotted, dashed and solid lines are 90 kg (Q1), 98 kg (Q2) and 107 kg (Q3) hinds body weight, respectively. (**b**) thin-magenta line: female calf; thick-black line: male calf; dotted, dashed and solid lines are hinds at age 3 years (Q1), 5 years (Q2) and 8 years (Q3) old. Triangle: male; circle: female.

The model clearly indicated that heavier hinds produced calves which ultimately grew heavier (asymptote estimate = 0.33, se = 0.050, *P* < 0.001, Table 1 in Supplementary Information 3), especially male calves, as pointed out by the significant interaction hind weight × calf sex in the asymptote parameter (calf estimate effect: 0.312, se = 0.071, *t*-value = 4.41, *P* < 0.001, Table 1 in Supplementary Information 3, Fig. 1a). Our model predicted weaning body weights of male and female calves raised by 90 kg hinds 3.1 and 2 kg lighter, respectively, than those of calves borne by 107 kg hinds (Fig. 1a). After accounting for the effect of hind weight the age of the hind had a negative effect on calf growth (asymptote estimate = − 1.73, se = 0.656, *P* = 0.009), and again this was especially pronounced for male calves, whose growth was hampered as their mothers got older, as compared with female calves’ growth (interaction hind age × calf sex [male], estimate: − 2.525, se = 0.981, *t*-value = − 2.57, *P* = 0.010,  Table 1 in Supplementary Information 3, Fig. 1b). At weaning male and female calves raised by hinds 3 years old were 5 and 2.7 kg heavier, respectively, than those predicted by male and female calves borne by hinds 8 years old (Fig. 1b). Male and female calves were heavier at weaning as parity increased (estimate: 1.828, se = 0.880, *t*-value = 2.08, *P* = 0.038, Table 1 in Supplementary Information 3, Fig. 1c).

### Descriptive statistics on milk yield and composition

**Figure 2:**
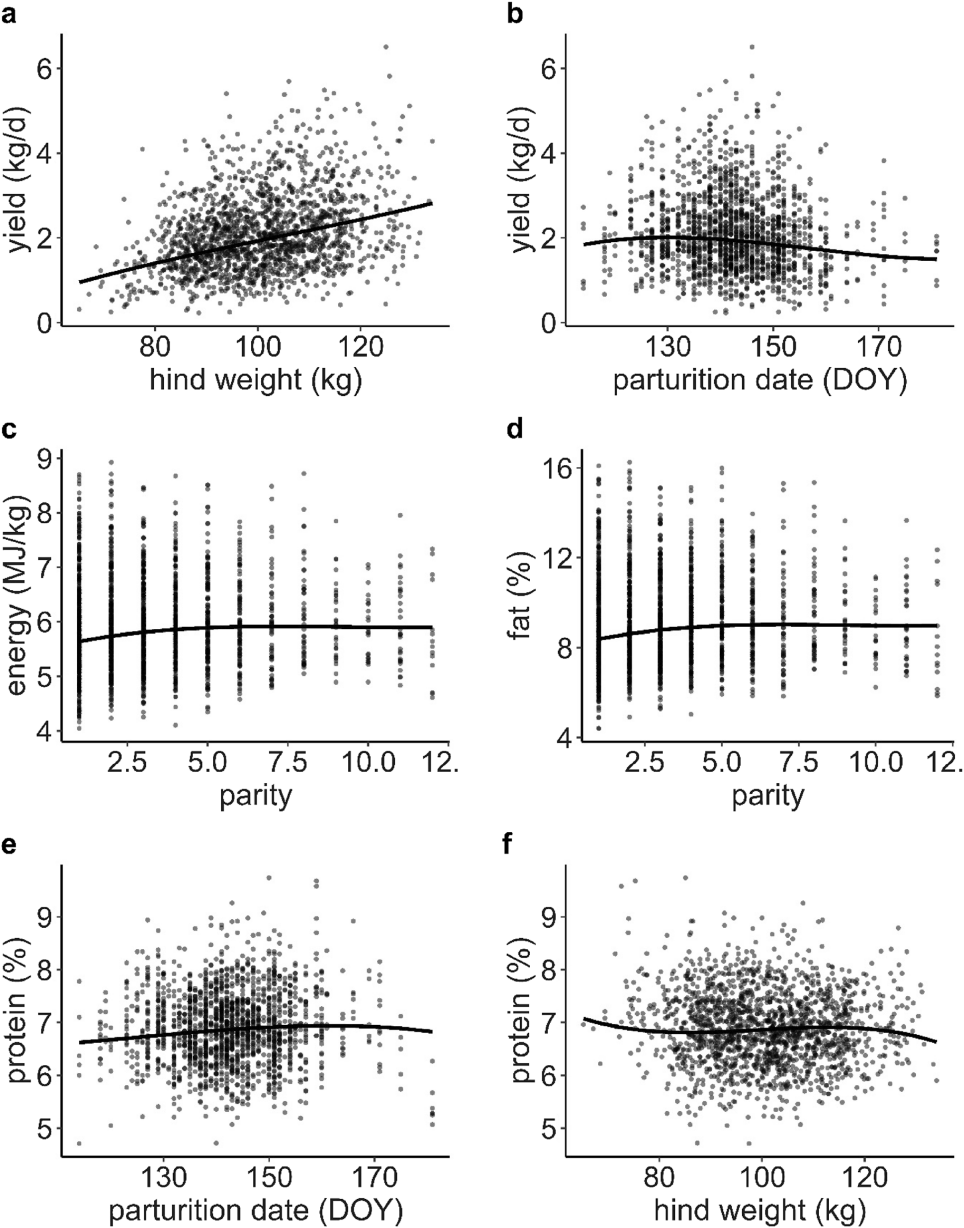
Predictions of the response of milk constituents against red deer hind traits using models in Supplementary Information 3 Tables 2–5.

Mean daily milk yield across lactation was 2.1 kg (Q1 = 1.4, Q3 = 2.6), mean composition was fat 8.9% (Q1 = 7.7, Q3 = 10.5), protein 6.7% (Q1 = 6.4, Q3 = 7.4), lactose 4.5% (Q1 = 4.1, Q3 = 4.9), and milk density energy 5966 kJ kg^-1^ (Q1 = 5346, Q3 = 6515, Fig. 1 in Supplementary Information 2). Daily milk yield decreased steadily as lactation advanced (Fig. 1 in Supplementary Information 2). Milk fat content remained constant for the first 60 days of lactation and increased afterward, as milk density energy did. A similar pattern was found in milk protein, but its increase across lactation was more attenuated. Lactose remained approximately constant across lactation. Mean calf weight at birth increased with hind age and hind weight up to 4 years of age and 100 kg of body weight, both traits remaining approximately stable when hinds got older or heavier (Figs. 2–3 in Supplementary Information 2). Daily milk yield clearly increased with hind weight, as did fat, energy, lactose and protein in milk with hind age.

### Milk yield

**Figure 3:**
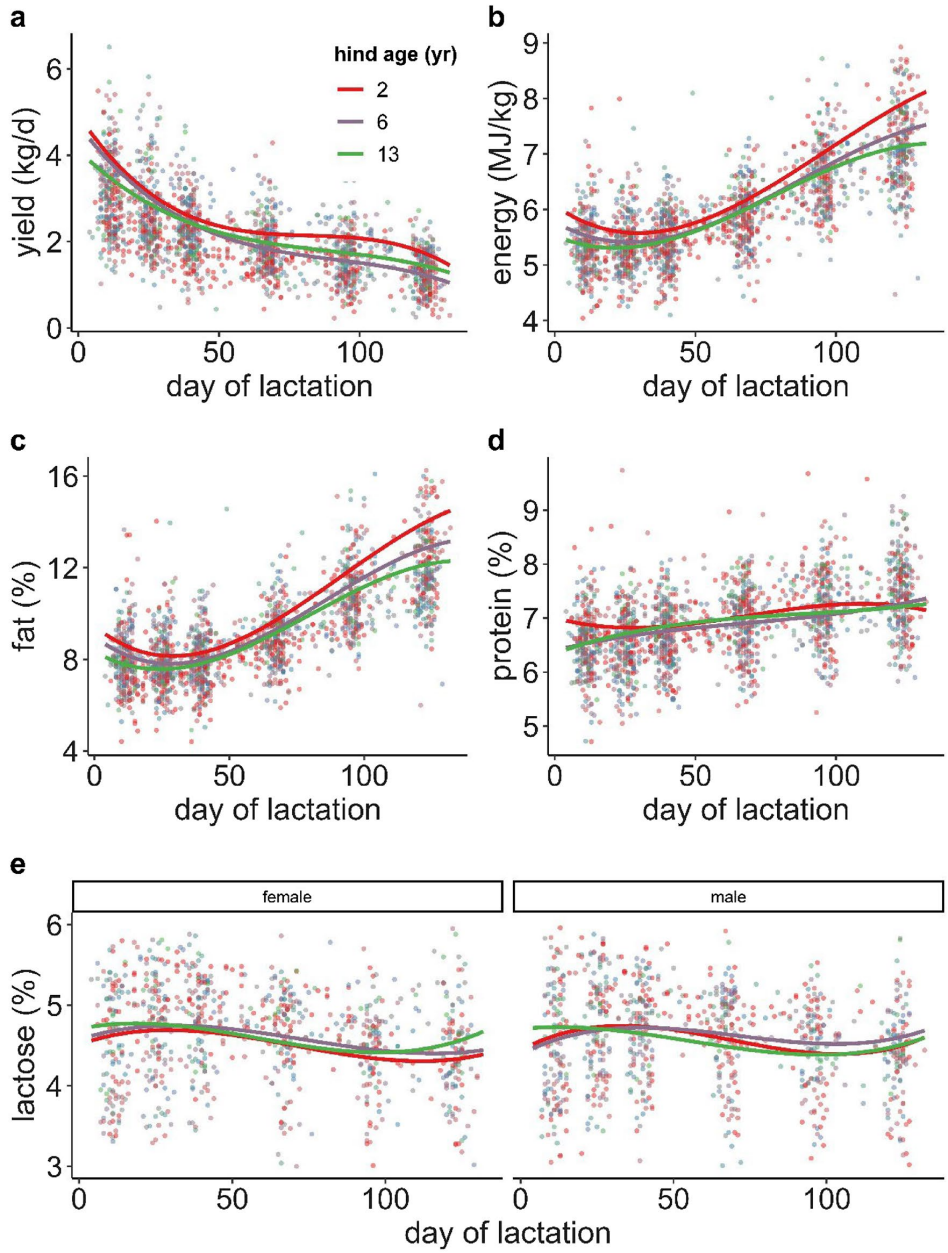
Predictions of the effect of red deer mother age on (**a**) milk yield (kg/d), (**b**) milk energy (MJ/kg), and (**c**) percentage of fat, (**d**) protein and (**e**) lactose in milk using models in Supplementary Information 3 Tables 2–6.

Model in  Supplementary Information 3 Table 2 predicted that the heavier the hind the greater her milk yield after controlling for all other terms in the model (Fig. 2a). Milk yield reached a maximum when calves were born around May 10th, while hinds with calves born earlier and especially later rendered lower milk yield (Fig. 2b). There was a complex interaction between hind age and day of lactation on milk yield (Fig. 3a). Milk yield decreased with day of lactation through the first 50 d of lactation especially in hinds up to 6 years of age (mean yield values: 2 years old = 3.6 kg d^−1^, 6 years old = 3.4 kg d^−1^, 13 years old = 3.2 kg d^−1^, Fig. 3a), on day 50 onwards the rate of decrease slowed down. By the end of lactation (day 130) the young hinds produced more milk than older hinds, and the very old hinds still produced more milk than younger adult hinds (2 years old = 1.5 kg d^−1^, 6 years old = 1.0 kg d^−1^, 13 years old = 1.3 kg d^−1^, Fig. 3a).

### Milk energy density

Milk energy was negatively affected by hind age, especially at the beginning and end of the lactation period, and increased through lactation in a cubic fashion (Fig. 3b, and Table 3 in Supplementary Information 3) (milk energy predicted means at the beginning of lactation were 5900, 5700, 5500 kJ kg^−1^ in hinds 2, 6 and 13 years old, respectively; and 8100, 7500, 7200 kJ kg^−1^ by the end of lactation). Milk energy density increased with parity from 5600 kJ kg^−1^ in primiparous hinds, and remained constant at 5900 kJ kg^−1^ beyond values of parity bigger than five (Fig. 2c, and Table 3 in Supplementary Information 3).

### Milk fat

There was a significant interaction between day of lactation and hind age in the response of fat content in milk (Fig. 3c), and the pattern of the response was very similar to that described for milk energy density (above), because of the large contribution that fat has on milk energy. Percentage of fat predicted means, at the beginning of lactation, were 9.1, 8.7, 8.1 in hinds 2, 6 and 13 years old, respectively; and 14.5, 13.1, 12.3 by the end of lactation (Fig. 3c). Fat increased with parity from 8.4% in primiparous hinds, and remained constant at 9.0% beyond values of parity bigger than five (Fig. 2d, and Table 4 in Supplementary Information 3).

### Milk protein

The predictions of the model (Table 5 in Supplementary Information 3) were that protein content increased from 6.6%, at the first date of parturition (24th April), up to 7.0% (10th June) and followed a slightly decreased until the last parturition date 6.8% (30th June, Fig. 2e). Predicted percentages of protein were higher in the lightest hinds (7.1%) than in the heaviest hinds (6.6%), with a predicted value of approx. 6.8% for most of the hind weight range (Fig. 2f). As day of lactation advanced, predicted percentage of protein in milk increased, but at different rates depending on hind age (Fig. 3d). Young hinds produced milk of higher protein content than older hinds, especially in the first 25 d of lactation (mean protein content in hind 2 years old = 6.8%, hind 6 years old = 6.6%, hind 13 years old = 6.6%) in comparison with the end of lactation (days 100 to 132, 7.2% in hinds 2, 6 and 13 years old).

### Milk lactose

Predictions from model in Supplementary Information 3 Table 6 indicated that lactose content in milk changed as lactation progressed, increasing in the first 25 days of lactation, followed by a decrease until day 100 and increasing later on. This general pattern interacted in a complex way depending on whether hinds raised female or male calves (Fig. 3e). At the beginning of lactation old hinds produced milk with slightly higher percentage of lactose than younger hinds regardless on the sex of the calf (4.7% vs. 4.5%). By the end of lactation the percentage of lactose was similar in old hinds lactating female or male offspring (4.7%), but younger hinds lactating female calves produced milk with lower content of lactose than those lactating male calves (4.4% vs. 4.7%, Fig. 3e).

### Senescence and differential allocation on calf growth and milk traits: literature data

Evidence of senescence in calf growth and milk traits is not consistent across studies and species (Table 2 and Material 2 in Supplementary Information 1), neither was there consistent evidence in the literature to support maternal differential allocation in milk traits (Table 3, and Material 3 in Supplementary Information 1). Out of 12 studies (Table 3) only five studies controlled for offspring weight, and of these, three found no differences in milk traits contingent on offspring sex, and one found no sex differences in one milk trait, and sex differences in other two milk traits (one as predicted by the theory and the other against prediction).

**Table 2:** Response of offspring growth and milk traits to mother age in published studies.

Source	Offspring growth	Fat %	Protein %	Carbohydrate %	Yield	Yield energy	Energy density	Age (yr)	Parity	Mother wt	Species
^27^	—	↓	↑[3]↓	—	—	—	—	—	1–5	?	*Bos taurus*
^28^	↑[5]↕^a^	—	—	—	↑[5]↕	—	—	2–10	—	?	*Bos taurus*
^22^	—	—	—	—	↑	↑	—	—	^b^	Yes	*Macaca mulatta*
^17^	—	—	—	—	↑ ^c^	—	ns ^b^	—	1–18	Yes	*Macaca mulatta*
^29^	—	—	—	—	↑ ^d^; ↑[4]↓ ^b^	—	—	1–8	1–5	No	*Bos taurus*
^30^	—	ns ^b, c^	ns ^b, c^	ns ^b, c^	—	—	—	—	—	No	*Homo sapiens*
^31^	—	—	↕[3]↓ ^e^; ns ^f^	—	—	—	—	2–≥ 6	—	No	*Bos taurus*
^32^	—	—	↑(7); ↓ ^h^; ns ^i^	—	—	—	—	2–≥ 6	—	No	*Bos taurus*
^10^	↑ ^j^	—	—	—	—	—	—	1–≥ 17	—	Yes	*Cervus elaphus*
^33^	ns	—	—	—	—	—	—	2—9	—	Yes	*Cervus elaphus*
This study	↓ ^d^; ↑ ^c^	↓ ^k^	complex ^k^	complex ^l^	↓ ^d^	—	↓ ^k^	1–≥ 17	1–12	Yes	*Cervus elaphus*

^a^Offspring weight at age 205 d; ^b^Primiparous vs. multiparous; ^c^Response to parity; ^d^Response to age; ^e^Significant for total protein and casein; ^f^Non-significant for serum protein; ^g^Inmunoglobulin; ^h^β-lactoglobulin or α-lactalbumin; ^i^Non-significant for bovine serum albumin; ^i^Growth rate; ^k^Interaction mother age × lactation day; ^l^Interaction mother age × lactation day × offspring sex.

Type of response: decreasing (↓), increasing (↑), plateau (↕). The age (and/or parity value) at which the response reaches a maximum or plateau is displayed in square brackets. Complex responses are depicted by a combination of arrows and number of years in brackets, for example, ↑[5]↕ represents an increase with age plateauing at 5 years. Non-significant (ns).

**Table 3:** Summary of studies in the literature that analysed milk provisioning, indicating the type of analysis, effects and covariates used.

Source	Species	Effects						Covariates						Analysis type			
								mother		offspring							
		Yield	Yield energy	Energy density	fat	Protein	Carbohydrate	Weight	age	Weight	age	Parturition	RH	Long/cross	n	Random	Non-linear
^20^	*Cervus elaphus*	♂ > ♀*	—	—	♂ = ♀	♂ > ♀*	♂ = ♀	✓^a^	—	✓	✓	—	—	L	4	Yr, IDm	—
^34^	*Ovis aries*	♂ < ♀	—	—	—	—	—	—	—	—	—	—	—	C	1	—	—
^21^	*Macaca mulatta*	—	♂ > ♀*	—	♂ > ♀*	♂ = ♀	♂ < ♀	ns	—	—	—	—	✓	C^b^	1	—	—
^22^	*Macaca mulatta*	♂ < ♀	♂ = ♀	♂ > ♀*	—	—	—	ns	—	✓	✓^c^	—	✓	C	1	—	—
^35^	*Bos taurus*	♂ < ♀	—	—	—	—	—	—	—	—	—	—	✓	L			
^23^	*Homo sapiens*	—	—	♂ > ♀*	—	—	—	—	—	—	—	—	—	C	> 1	—	—
^24^	*Homo sapiens*	—	—	—	♂ > ♀	—	—	✓^e^	✓		✓	—	✓	C	5	—	—
^36^	*Homo sapiens*	—	—	♂ > ♀*	♂ > ♀	—	—	—	—	—	✓	—	—	C			
^37^	*Homo sapiens*	—	—	♂ = ♀	♂ = ♀	♂ = ♀	♂ = ♀	✓	—	✓	✓	—	ns	C			
^25^	*Macropus eugenii*	—	—	♂ = ♀	♂ = ♀	♂ > ♀*	♂ = ♀	✓	—	—	—	—	—	C	2^d^	IDm	—
^38^	*Zalophus californianus*	♂ = ♀	—	—	—	—	—	—	—	✓	✓	—	—	C	1?	—	—
This study	*Cervus elaphus*	♂ = ♀	—	♂ = ♀	♂ = ♀	♂ = ♀	♂ vs ♀^f^	♂ = ♀	✓	✓	✓	✓	✓	L	5–14	Yr, IDm IDo	✓

^a^Mother weight after giving birth, it does not account for mother weight changes across lactation. ^b^Primiparous vs multiparous. ^c^Age at peak of lactation, this is only one age record within offspring. ^d^Two phases of lactation. ^e^Body condition index. ^f^Complex interaction. *Supporting differential allocation of resources offspring sex-dependent.

Yield: milk yield; yield energy: energy provided by milk yield; fat, protein and carbohydrate content in milk; weigth: body weight; parturition: parturition date; RH: maternal reproductive life history (parity); long/cross: longitudinal (L) or cross-sectional (C) population analysis; n: number of milking events across lactation; random: random effects when a linear mixed model was used; IDo: offspring ID; IDm: mother ID; Yr: year effect; ♀: daughter; ♂: son; ✓: variable included in the analysis; —: variable not included in the analysis. Notes in brackets.

## Discussion

The results of this study indicate that: (i) calf growth and milk yield, energy density and fat content were negatively affected by hind age in old mothers, and improved with parity except in yield, (ii) milk yield was positively correlated with hind weight, and (iii) milk yield and protein content peaked at specific parturition dates. The results therefore, in general, support predictions P1, P2, P3 and P6 (Table 1, and Material 1 in Supplementary Information 1). Maternal differential allocation biased to males was found in calf growth, supporting prediction P5, in agreement with differential allocation theory, and growth in sons was more negatively affected by hind age and more positively affected by hind weight than the growth in daughters. All milk traits, but lactose, showed no differential allocation between offspring of different sex, and in the case of lactose the pattern of differential allocation found was difficult to interpret.

The effects of aging on milk traits and offspring growth varies across studies and makes difficult to draw conclusion because of differences in statistical modelling, not controlling for confounded factors, and not having a sufficient sample of the oldest age classes, the latter being crucial if aging effects occur only very late in life (Table 2), as pointed out by Nussey and collaborators^11^ in a wild population of red deer.

Our results indicate that calf growth is offspring male-biased and affected by hind age (negatively in the old age classes) and hind condition (positively), which supports one premise of the differential allocation theory^5^, that resources provisioning from mother to offspring should have a greater effect on growth and fitness of sons than those of daughters of highly polygynous species^3^. This sex-biased growth was not due to a corresponding sex-biased higher maternal allocation in milk constituents, after calf body weight was taken into account. This suggests that in our hinds resource provisioning responded to offspring energy requirements, conditioned to mother age and weight, and intrinsic offspring sex-specific modulation of growth, as suggested in macaques by Hinde^22^.

The two main regulatory channels of milk yield and composition are the mammary gland and blood plasma, the latter is mainly affected by dietary and digestive factors (Material 4 in Supplementary Information 1). Any behavioural and aging processes that affect these two channels have a detrimental effect on milk traits. In our hinds, milk yield, fat and energy density were the traits with the largest variation through lactation, followed by protein, with lactose remaining approximately constant. This corroborates the findings of other studies in ruminants^16,39^. Lactose suffers minimum variation through lactation, because of its role as osmotic regulator in the mammary cells^16^, which might explain the complex interaction that we found in the lactose response to calf sex across lactation.

Milk energy provisioning to the offspring contingent on the mother´s age could be offset via milk yield and/or milk quality, although what should be the optimum contribution of each to maximise offspring growth is unclear, as pointed out by Hinde et al.^22^. The problem lies in the fact that milk traits are somehow correlated, especially fat and protein^21^. In macaques, milk energy density and milk yield increased as lactation progressed but there was a trade-off, as those mothers with a greater increase in milk energy density had a smaller increase in milk yield, with a concomitant decrease in their offspring growth^17^. In cattle, milk yield and fat concentration are negatively correlated, and genetic selection that increases the percentage of protein in milk frequently decreases milk yield^16^. In milk from macaques carbohydrates are negatively correlated with milk fat^21^. Our results indicate that hind age has a negative effect on milk fat, and consequently on milk energy, both traits decreased as hinds get older. This finding contrasts with taxa whose milk is low in energy, such as in primates and humans, where a good predictor of offspring growth is milk yield, but not energy density^17,40^.

There is a plethora of evidence that dietary and foraging factors that influence nutrients availability and their digestion affect milk traits. For example, increasing dietary protein and dietary energy has a stronger positive effect on milk yield than on milk protein concentration^27,41^. In our hinds any process of regulation of milk yield and composition via diet selection were likely to be limited as there was little dietary choice.

Parity can be understood as a proxy of reproductive experience across the mother´s life. We found that milk fat had a positive asymptotic response to parity (reaching a plateau at 6 successful calving seasons), while calf growth improved in a linear fashion across the mother´s reproductive life. There are trade-offs between the mother´s reproductive senescence and reproductive experience on reproductive allocation of resources. Maternal care improves through an animal´s reproductive life by developing a repertoire of behaviours, based on previous experience, that favour offspring growth and survival with mother age^3^. This behavioural repertoire might also affect milk traits in situations where mothers compete for food or when foraging strategies are possible^42^, something that is unlikely to happen in well-fed captive deer, as in our case.

Reproductive allocation has been related to the mother´s body reserves^22^. Primiparous females are supposed to have less fat reserves than multiparous, and so milk yield and milk energy density is expected to be higher in multiparous females, which can be confounded with maternal reproductive experience. Findings across studies are not consistent, primiparous mothers of baboons and gorillas^43,44^ had poorer reproductive outcome than experienced mothers; milk yield and energy density was higher in primiparous than in multiparous mothers of Rhesus macaques^22^. In the same species Hinde et al.^21^ found a significant interaction between parity and offspring sex in offspring weight at 3–4 months of age, daughters of primiparous mothers were lighter than daughters of multiparous mothers and also lighter than any son. These authors claimed significant interactions between offspring sex and parity in energy, fat and protein milk traits of the mother. For example, milk protein was higher in offspring males of primiparous mothers than in any other offspring of primiparous or multiparous mothers. The interpretation of these results requires caution, as it seems that sex and offspring weight were confounded in their analyses. Our findings on parity (i.e., positive response of milk energy, fat and calf growth) corroborate those of other authors. Hinde and collaborators^21^ found that primiparous mothers produced lighter offspring than multiparous mothers. Landete-Castillejos and collaborators^45^ reported that red deer yearling mothers gave birth to lighter calves, lower milk yield and calf growth rates than those produced by subadult and adult mothers.

One of the most tested theory on parental care is the differential allocation theory^4,5^, but support to this theory in milk traits is weak and varies across studies and species (Table 3). The mechanisms by which mothers might regulate milk yield and composition dependent on the sex of their offspring are unknown, which opens up the interpretation of results across studies to speculation. Hinde^22^ attributes the discrepancy of results between studies to species, differences in life history strategies, ecological pressures, metabolic efficiency, anabolic regulation, growth hormones and milk assimilation^38^. We highlight that some differences between studies are merely due to differences in statistical modelling, mainly, poor control of confounded variables and restricting the response to linearity (Table 3). One of the potential mechanisms put forward to explain how mothers might modulate milk yield and composition in an offspring sex-dependent manner is hormonal signalling^46^. Mammary gland development could be affected during pregnancy by exposure to foetus testosterone, which in presence of estrogen synthase produces estradiol that might promote fat content in milk. The dependence of milk yield energy on maternal age has been associated with differences in sensitivity to foetal hormones of endocrine systems of immature and adult mothers^22,46^. Whether hormone signalling is one or the ultimate mechanism in the modulation of milk traits to offspring sex and mother age remains elusive.

Are there other extrinsic factors that could explain the absence of differential allocation effects in our study? In the same population used in this study it was observed that as the mother´s milk yield decreased, the number of allosuckling events of sons increased but remained constant in daughters^47^, unfortunately, this study does not provide information of the total energy contribution of allosucking across lactation, which makes difficult to draw any conclusion.

We found that parturition date did not influence calf growth. There are studies that found that early births produced faster growth in captive deer calves^48,49^. Some of these studies used data in which calving season was artificially advanced^48^, which obviously enhanced the effect of parturition date on offspring growth. In wild populations of red deer early births have been associated with those corresponding to dominant mothers, resulting in higher rates of offspring winter survival^50^. There is evidence that supports that parturition date is adaptive, probably as a mechanism to maximise offspring growth by synchronising parturition to favourable conditions for plant growth. In a wild population of red deer Moyes and collaborators^51^ found that average parturition date suffered an advance of 12 days across a 28 years period and it was associated with plant growth phenology. In our animals, food resource availability was unlikely to be associated with phenology, as they did not rely on grazing, which could explain why we did not find any relationship between calf growth and parturition date. Year of lactation had an important effect on calf growth and milk traits, which might be partially due to variation in abiotic conditions across years that affects plant growth^52^, diet quality and composition. In captive animals, as in our case, this effect is less relevant than in wild populations, but still important. Although we tried to provide deer with a consistent diet across years, annual differences in forage supplied were inevitable. In a long-term longitudinal study on a captive population of Iberian red deer, Pérez-Barbería et al.^10^ found that under low heat years calves grew up 1.2 kg heavier at weaning than those growing in high heat years, and males were more affected by heat stress than females.

We found that only milk yield and milk protein responded to parturition date, peaking on 10th of May and 10th of June, respectively (mean parturition date = 23rd of May). It is interesting that no response was found in milk fat content, which suggests mother-intrinsic mechanisms of milk protein provisioning regulation as a response to calf growth requirements.

It has long been recognised that there is high inter-individual variability in milk constituents^39^. Consistently, our results show important levels of hind variance in most milk traits. It could be that hinds differed in ruminal microbiota^53^, although it seems unlikely, as they share the same diet and biotic environment, or that they responded to individual offspring energy demands, the mechanisms involved are unknown. High milk production in cattle and sheep has been possible through individual selection for milk traits, which points out a high individual variation heritability^54^. We found that milk yield was the only milk trait that some of its variation was explained by mother of the hind, which suggest the importance of maternal environment in milk yield in captive red deer.

## Methods

### Study area and animals

Data collection was carried out at the University of Castilla-La Mancha (UCLM) experimental small ruminant farm in Albacete (Spain) between 1998 and 2020 (Material 5 in Supplementary Information 1). The study used 156 hinds of 22 cohorts and 635 calves (Table 7 in Supplementary Information 3). The mean number of monitored calves per season was 29 (sd = 20.3) and lactating hinds were milked between 1 and 12 lactation seasons (mean = 7, sd = 8.2, Table 8 in Supplementary Information 3).

### Animal and milk traits

Age of deer was accurate within 1 day as all births were monitored, recording date, time and calf weight. Calf age was in days and age of hinds was transformed into 17 year classes (1 up to ≥ 17 years). Parity was defined as the summation of the number of calves at a given mother age. Milking took place at, approximately, day of lactation 9, 27, 40, 67, 98 and 125 (Fig. 4 in Supplementary Information 2, Material 6 in Supplementary Information 1). Milk yield was defined as milk production (kg) across 24 h and was calculated based on one milking event over a period of 6 h^55^ (Material 7 in Supplementary Information 1). Milk composition was analysed using standard spectrophotometry techniques (Milkoscan FT6000, Foss Electric, Hillerød, Denmark). Concentration of fat, protein and lactose were expressed as weight percentage of milk. Calculation of milk energy was carried out following Perrin´s Equation^56^. The experiment was reviewed by the Animal Welfare and Ethical Review Body of the scientific establishment (Comité de Ética en Experimentación Animal CEEA, University of Castilla-La Mancha, no. PR-2021–09-18), and the study is reported in accordance with ARRIVE guidelines (https://arriveguidelines.org) and complied with Spanish regulations on animal experimentation (Law 32/2007 7th of November; Royal Decree 53/2013 1st Feb; ECC/566/2015).

### Statistical analysis

We used GAMM models (Generalized additive mixed models) as an exploratory approach, implemented in the “*gam”* function of the mgcv R package^57^, followed by linear mixed models with polynomial functions equivalent to those obtained by the GAMM models, implemented in the package lme4^58^ in R software version 3.4.1^59^. Calf growth was modelled fitting an exponential curve, parameterised using non-linear mixed regression models implemented in the R package nlme^60^. To improve the readability of the models’ output we plotted the response of interest by fixing the other explanatory variables to their mean values or to convenient quantiles. Graphics were constructed using the ggplot2 R package based on The Grammar of Graphics^61^. For details on the statistical analyses see Material 8 in Supplementary Information 1.

## Conclusion

This study and literature review revealed that there is little evidence for the differential allocation theory on milk traits. Calf growth is male-biased, negatively affected by mother age and positively influenced by mother condition and parity. Our results indicate that controlling for maternal and offspring factors and allowing for non-linear responses is crucial for assessing the effect of senescence and differential allocation on milk traits contingent upon offspring sex.

## Supplementary Information

Supplementary Information 1.

Supplementary Information 2.

Supplementary Information 3.

## Data Availability

The datasets used during the current study available from TL-C on reasonable request.
