# Supplementary material for: Effects of maternal age and offspring sex on milk yield, composition and calf growth of red deer (Cervus elaphus)
Source: Sci Rep. 2022 Aug 25;12:14506. doi: 10.1038/s41598-022-17978-3 (PMC9411626; doi:10.1038/s41598-022-17978-3)
Supplement: Supplementary file 1 — Supplementary Information 1. [file 41598_2022_17978_MOESM1_ESM.docx]

**Material 1. Predictions.**

In line with predictions from classical theory ^1–4^ we expect (P1) that milk yield, milk energy density, milk composition and calf growth will respond to mother age following this pattern: improvement through early life, reaching a plateau in prime age, and then suddenly decline. P2. milk yield, milk energy density, milk composition and calf growth will improve as parity increases reaching a plateau in prime age, followed by a maintained gradual decline, that is favoured because of the advantages of reproductive experience, and then a sudden decline when reproductive experience cannot compensate for the deterioration of physiological function. P3. A general increase in milk yield, milk energy density, milk composition and calf growth with mother condition. P4. Milk yield is produced accordingly to the energy requirements (size) of the offspring, consequently, milk yield will be greater in mothers lactating male offspring than those lactating female offspring; P5. Milk yield, milk energy density and milk composition will not differ between offspring sexes (after controlling for offspring weight), this is because, (i) milk yield is produced accordingly to the energy requirements (size) of the offspring, and (ii) milk energy density and composition are mainly driven by diet composition ^5^ and in our captive deer there is very little choice for mothers to select diets that enable them to modify milk composition. P5. Mothers in captive conditions that have access to *ad libitum* food will attempt to satisfice their offspring milk demand, consequently, offspring growth will be greater in males and in females of dimorphic species in body mass. P6. Early parturition dates generally correspond with mothers in good condition but also with synchronising lactation with vegetation growth, in well fed captive deer which diet is not dependent on natural plant growth conditions, parturition dates might have little effect on milk yield, milk energy density, milk composition and calf growth.

**Material 2. Evidence of senescence in reproductive traits.**

Evidence of senescence in calf growth and milk traits is not consistent across studies and species (Table 2). Some studies pointed out an increase in milk yield and milk yield energy with mother´s age in Rhesus macaque *Macaca mulatta* ^6,7^, no demise in human milk composition with mother age ^8^, evidence of maternal aging effect in offspring growth and milk constituents (fat, protein, yield, density energy, this study), mother aging effect in some serum proteins of cow milk but not in other milk proteins ^9,10^, an increase with maternal age that reached a plateau in milk yield and calf growth in cattle ^11^, increase followed by decline in the concentration of milk constituents, milk yield and milk energy density in cattle and red deer (in ^12,13^, and this study).

**Material 3. Evidence of differential allocation in milk traits.**

There was no consistent evidence in the literature to support maternal differential allocation in milk traits (Table 3). One study found that red deer hinds raising male calves produced higher milk yield than hinds bearing female calves ^14^. In Rhesus macaque ^6^ and sheep (*Ovis aries*) [not controlling for calf weight ^15^] the opposite was found, milk yield was higher in mothers raising female offspring. In Californian sea lion (*Zalophus californianus*) ^16^ and red deer (this study) no differences in milk yield between females bearing offspring of different sex were found after controlling for offspring weight. In macaque yield milk energy ^17^, milk energy density ^6^ and milk energy density in humans ^18^ were higher in mothers bearing male offspring in comparison with milk of mothers bearing female offspring; no differences contingent on offspring sex were found in milk yield energy ^6^ or in energy density in Tammar wallaby *Macropus eugenii* ^19^ or in yield energy and energy density in red deer (this study). Three studies show no offspring sex-related differences in fat content (Landete-Castillejos et al. 2005, Robert and Braun 2012, this study) neither in protein content ^17^, and there were no indications of sex differences in milk carbohydrates concentration ^14,19^, though Hinde ^17^ found female offspring milk being higher in carbohydrates that male offspring milk. All in all, out of 12 studies (including this) that tested the differential allocation theory (Table 3) only five studies controlled for offspring weight, and of these, three found no differences in milk traits contingent on offspring sex, and one found no sex differences in one milk trait, and sex differences in other two milk traits (one as predicted by the theory and the other against prediction).

**Material 4. Physiology of milk production.**

Following Linn ^5^, the predominant milk fat constituent is triacylglycerol, about 50 % of its fatty acids, those of short-chain length, are synthesized in the endoplasmic reticulum of the mammary alveolar cells, long-chain acids are derived from blood plasma fatty acids, and intermediate-chain acids come from both sources. The main constituent of milk protein is casein (approx. 80 %), the rest are serum and whey proteins, most of these proteins are synthesized in the rough endoplasmic reticulum and Golgi apparatus of the mammary gland cells, via DNA control standard protein synthesis mechanisms. The principal carbohydrate in milk is lactose ^20^, a disaccharide formed in the Golgi apparatus and vesicles of the mammary gland cells, using glucose and galactose that 85 % is derived from blood plasma. Its main function is to maintain the osmotic equilibrium in the vesicles with surrounding intra-cell fluids, via water flow though the vesicle membrane, making lactose the chemical constituent that regulates milk yield ^21^. Consequently, the two main regulatory channels of milk yield and composition are the mammary gland and blood plasma, the latter is mainly affected by dietary and digestive factors. Any behavioural and aging processes that affect these two source channels have a detrimental effect on milk traits.

**Material 5. Farm facilities and animal feeding.**

The UCLM farm is a scientific facility oriented to the study of life history traits, reproduction, nutrition and antler growth in Iberian red deer. Deer groups lived in 0.5 ha fenced fields with access to drinking water and food. The main diet was a mixture (80% - 20%) of chopped alfalfa hay and orange pulp ^22^ supplied *ad libitum* three times a week, together with a well-balanced mineral supplementation. Between March and October this diet was supplemented with a commercial pelleted feed (crude protein = 17%, fibre = 10.2%, fat = 3.2%, ash = 9.8%). Food was presented to deer on both-side access 14 m long belt feeders to minimise aggressions during feeding and reduce competition. This experimental setup allowed us to minimise the effect of bullying during food access that otherwise could negatively affect the condition of old hinds. Dedicated personnel attended the animals and a veterinarian checked them on a weekly basis.

**Material 6. Body weight monitoring and milking.**

Within lactation cycle (approx. 134 d) hinds were milked between 5 and 14 times (mean = 6.2, sd = 3.36). The number of milking monitoring events varied across years for logistical and experimental reasons, but also it was intended to minimise stress and disruption of the hind-offspring bond, while still allowing a reasonable sample size to detect changes in milk yield and milk composition through lactation (Landete-Castillejos et al., 2000; Robbins, 1993, p. 203). At each monitoring event deer were driven from the fields to the handling facility through a system of alleys, where they were weighed on a plate platform scale fitted with a motion hold sensor (± 0.05 kg). Calves at birth were weighed in the field, in close presence of their mothers, using a digital portable scale (± 0.01 kg). Hind weight was missing in a few monitoring events, in those cases we used the hind weight of the closest available monitoring date within 8 days. Calves that did not survive 59 days were excluded from the analysis, this was not a source of sex bias as calf mortality was similar between sexes. Before milking lactating hinds were separated from their calves for 6 hours and to reduce stress mother and calves were in adjacent group-pens in close visual contact. During this period, hinds did not have access to food or water. Milking was carried out with an initial brief manual massage stimulation of the udder followed by conventional application of a milking machine, designed for goats, with vacuum pulsation chambers (44 kPa) and transparent teatcup shells 25. Each teat was milked individually between 30-60 s until milk stopped flowing into the teatcup, and milking was finished by hand. Total milk yield was measured in a graduated cylinder and kept refrigerated at 4°C until chemical analysis (see below). The negative effect that stress during milking has on milk yield is well-known, especially in semi-domesticated species 26–28. To minimise stress, milking was carried out under sedation with a low-dose of a mixture of xylazine and ketamine hydrochloride (0.5 mg kg-1, 1 mg kg-1 body weight, respectively) delivered by intramuscular injection. Once sedation was taking effect, milk letdown was induced by 10 IU of exogenous oxytocin injected in the jugular vein 1 min before starting milking, and immediately after milking the effects of anaesthesia were reversed via a jugular yohimbine injection 0.25 mg kg-1 body weight 24. Hinds recovered from the anaesthesia in a group pen, and were inspected before being released into the fields in the company of their offspring. We did not observe post-milking adverse effects of the anaesthesia in hinds’ behaviour or effects on the mother-calf bond. The procedure was undertaken by a qualified veterinarian. Sedatives and oxytocin have been used successfully on marsupials 19, ungulates 14,29 and non-human primates 6. Some hinds were kept at the farm during all their reproductive life, and consequently, when their condition deteriorated rapidly and continuously for two weeks, were slaughtered, using an overdose of pentobarbital. This might have caused some selective mortality in our dataset, which could not be controlled for in our analyses as the consistency of monitoring these events across the study period varied.

**Material 7. Milk composition.**

Estimation of daily milk yield assumes that (i) milk collected in a milking event (see SEM 3) was representative of the milk produced in 6 h across a 24 h period, and (ii) the time between the last sucking event and the start of the period in which hind and calf were separated was the same across all mother-calf dyads. Although these assumptions were unlikely to be met, many studies have used a similar protocol to estimate milk yield successfully ^23^. Furthermore, the potential effect of not meeting assumption (ii) on the estimate of milk yield is minimised by the fact that milk accumulation in the udder reduces milk production ^30^. The length of milking interval also affects milk composition, especially fat, but has a minor effect, if any, on protein and lactose concentration ^5,18^. To convert milk volume into milk weight we used an average milk density of 1.038 g/ml that was measured from the milk of our deer ^31^. Milk of domestic ruminants and camels contain about one-half the protein, fat and energy of wild ungulate species´ milk ^23,32,33^. Consequently, to calculate milk composition applying standard analytical techniques of the dairy industry laboratories, milk collected was diluted in distilled water by a factor of two. Two replicates, per hind and milking event, of 15 ml of diluted milk were refrigerated and submitted to the laboratory within 24 h of milking. Milk composition was analysed using standard spectrophotometry techniques (Fourier Transform InfraRed) compliant with International Dairy Federation and Association of Official Analytical Chemists. The analyses were carried out in duplicate at CENSYRA laboratory between 1998-2010 (Valdepeñas, Spain) using a Milkoscan 500 (Foss Electric, Hillerød, Denmark), and at LILCAM laboratory between 2011-2020 (Talavera de la Reina, Spain) using a Milkoscan FT6000. Methods and calibrations followed International Standards ISO 8196-2/IDF 128-2 (2009) and ISO 9622/IDF 141 (2010). Concentration of fat, protein and lactose were expressed as weight percentage on undiluted milk. Calculation of milk energy was carried out following Perrin´s equation ^34^ that uses specific calorific values of fat, protein and lactose.

**Material 8. Statistical analysis.**

We conveniently divided our variables into two data sets, one containing information on milk traits (milk composition: percentage of fat, protein and lactose; milk yield kg d^-1^, milk density energy kJ kg^-1^), and a second one comprising information on calf growth (calf body weight, kg) during lactation. The analysis on milk traits used predictor variables associated with the hind (age, body weight, parity, parturition date) and its offspring (sex, age, body weight). Parturition date was transformed into day of year, where day = 0 was 1^st^ of January. Attempts at including primiparous status of the hind in the analysis (Hinde 2009) produced abstruse results, essentially because it was confounded with parity; consequently, it was not included in the final analysis. We also attempted fitting parity by calf sex, but this produced a sparse data matrix, therefore parity was calculated using the number of the calves of both sexes.

As an exploratory approach, we used GAMM models (Generalized additive mixed models with multiple smoothing parameter estimation by restricted marginal likelihood), implemented in the “*gam”* function of the mgcv R package ^35^. The model showed that the GAM smooth relationships were in fact generally quite simple and could be well-described by simple polynomial functions. Consequently, we used linear mixed models with polynomial functions equivalent to those obtained by GAM models, implemented in the package lme4 ^36^ in R software version 3.4.1 ^37^. We restricted the polynomial functions to degree = 3, as this was appropriated to test our predictions and a higher degree makes difficult the interpretation of interactions. Significance of the terms in the model was assessed using the R function lmerTest ^38^ that approximates degrees of freedom via Satterthwaite's method, as in linear mixed-effects models degrees of freedom are difficult to define appropriately ^39^. The variance explained by the linear mixed model was represented as *R^2^* marginal (variance accounted for by the fixed effects) and *R^2^* conditional (variance accounted for by random and fixed effects), following a method developed for linear mixed-effects models ^40^. Polynomial models on milk traits included age and weight of the hind, parity, parturition date, calf weight, lactation day and the random effects calf, hind and mother of the hind identities and year. Mother of the hind identity represented the identity of the mother of the hind that was monitored, and was included in the analysis to assess the effect of the maternal environment on milk traits. In order to not over-parameterise the models, only first order interactions of all fixes effects against calf sex, and two second order interactions (hind weight x lactation day x calf sex, hind age x lactation day x calf sex) were fitted. Hind age and parity were highly correlated, but this did not cause a large increase in the estimates of standard errors or variances (for fixed or random effects) in the modelling, consequently we retained parity in our models as it was interesting for comparative purposes against other studies from the literature. The full models were simplified using backward elimination by removing the non-significant fixed-effects terms, one at a time, following the principle of marginality (i.e. the highest order interactions were tested first and if they were significant, then the lower order effects were not tested for significance). Backward elimination was based on p-values in favour of information theory approaches ^41^, as successfully used in Pérez-Barbería et al. ^42^ and Carranza et al. ^43^.

It is widely accepted that calf growth fits an exponential curve, for which parameters can be easily interpreted ^44–46^. This curve can be parameterised using non-linear mixed regression models implemented in the R package nlme ^47^. We followed Pérez-Barbería et al ^48^ and evaluated an exponential regression function and its gradient of the type,

$f\left( x \right)=Asym+\left( R0-Asym \right)\times e^{{-e}^{lrc}\times t}$ Eq. 1

where *t* is day of lactation; *Asym* represents the horizontal asymptote; *R0* is the response at *t* = 0; and *lrc* is the natural logarithm of the rate constant, reflecting how quickly the fitted curve approaches the asymptote. This model included the following fixed effects: age and weight of the hind, parity, parturition date and sex of the calf. Because we were interested in assessing how calf growth was affected by sex, we fitted first order interactions with calf sex. The only random effects were the intercepts and we did not include neither hind identity nor year as the would both have been strongly aliased with hind-specific data in the fixed effects. The starting values of the fixed effect parameters of the full mixed effect model were estimated using a simpler model ignoring the random effect structure, implemented in the nonlinear least-squares regression package (nlm) of the R software. Finally, the estimated parameters were used as starting values to fit the full model with the addition of the random effects, and the model was re-run to corroborate parametrisation consistency and algorithm convergence.

**References**

1. Clutton-Brock, T. H. Reproductive effort and terminal investment in iteroparous animals. *Am. Nat.* **123**, 212–229 (1984).

2. Kruuk, L. E. B. *et al.* Antler size in red deer: Heritability and selection but no evolution. *Evolution* **56**, 1683–1695 (2002).

3. Nussey, D. H., Kruuk, L. E., Donald, A., Fowlie, M. & Clutton-Brock, T. H. The rate of senescence in maternal performance increases with early-life fecundity in red deer. *Ecol. Lett.* **9**, 1342–1350 (2006).

4. Nussey, D. H. *et al.* Inter- and Intrasexual Variation in Aging Patterns across Reproductive Traits in a Wild Red Deer Population. *Am. Nat.* **174**, 342–357 (2009).

5. Linn, J. G. Factors Affecting the Composition of Milk from Dairy Cows. in *Designing Foods: Animal Product Options in the Marketplace* (National Academies Press (US), 1988).

6. Hinde, K. Richer milk for sons but more milk for daughters: Sex-biased investment during lactation varies with maternal life history in rhesus macaques. *Am. J. Hum. Biol.* **21**, 512–519 (2009).

7. Hinde, K., Power, M. L. & Oftedal, O. T. Rhesus macaque milk: magnitude, sources, and consequences of individual variation over lactation. *Am. J. Phys. Anthropol.* **138**, 148–157 (2009).

8. Jenness, R. Biochemical and nutritional aspects of milk and colostrum. in *Lactation / edited by Bruce L. Larson ; written by Ralph R. Anderson ... [et al.]* 164–197 (Iowa State University, 1985).

9. Ng-Kwai-Hang, K. F., Hayes, J. F., Moxley, J. E. & Monardes, H. G. Environmental Influences on Protein Content and Composition of Bovine Milk. *J. Dairy Sci.* **65**, 1993–1998 (1982).

10. Kroeker, E. M., Ng-Kwai-Hang, K. F., Hayes, J. F. & Moxley, J. E. Effect of β-Lactoglobulin Variant and Environmental Factors on Variation in the Detailed Composition of Bovine Milk Serum Proteins. *J. Dairy Sci.* **68**, 1637–1641 (1985).

11. Lubritz, D. L., Forrest, K. & Robison, O. W. Age of Cow and Age of Dam Effects on Milk Production of Hereford Cows. *J. Anim. Sci.* **67**, 2544–2549 (1989).

12. Rogers, G. & Stewart, J. The effects of some nutritional and non-nutritional factors on milk protein concentration and yield [dairy cattle]. *Aust. J. Dairy Technol.* 26–32 (1982).

13. Khan, M. S. & Shook, G. E. Effects of Age on Milk Yield: Time Trends and Method of Adjustment. *J. Dairy Sci.* **79**, 1057–1064 (1996).

14. Landete-Castillejos, T., García, A., López-Serrano, F. R. & Gallego, L. Maternal quality and differences in milk production and composition for male and female Iberian red deer calves (<Emphasis Type="Italic">Cervus elaphus hispanicus</Emphasis>). *Behav. Ecol. Sociobiol.* **57**, 267–274 (2005).

15. Abecia, J. A. & Palacios, C. Ewes giving birth to female lambs produce more milk than ewes giving birth to male lambs. *Ital. J. Anim. Sci.* **17**, 736–739 (2018).

16. Ono, K. A. & Boness, D. J. Sexual dimorphism in sea lion pups: differential maternal investment, or sex-specific differences in energy allocation? *Behav. Ecol. Sociobiol.* **38**, 31–41 (1996).

17. Hinde, K. First-time macaque mothers bias milk composition in favor of sons. *Curr. Biol.* **17**, R958–R959 (2007).

18. Powe, C. E., Knott, C. D. & Conklin‐Brittain, N. Infant sex predicts breast milk energy content. *Am. J. Hum. Biol.* **22**, 50–54 (2010).

19. Robert, K. A. & Braun, S. Milk Composition during Lactation Suggests a Mechanism for Male Biased Allocation of Maternal Resources in the Tammar Wallaby (Macropus eugenii). *PLoS ONE* **7**, (2012).

20. Oftedal, OT. Milk composition, milk yield and energy output at peak lactation : a comparative review. *Symp. Zool. Soc. Lond.* **51**, 33–85 (1984).

21. Akers, R. M. Overview of Mammary Development. in *Lactation and the Mammary Gland* 3–44 (John Wiley & Sons, Ltd, 2016). doi:10.1002/9781119264880.ch1.

22. Habeeb, Gad, A. E., Mustafa, M. M. M., Atta, M. A. & Basuony, H. A. M. Using of Citrus By-Products in Farm Animals Feeding. (2017) doi:10.32628/IJSRST173681.

23. Robbins, C. T. *Wildlife feeding and nutrition*. vol. 2nd (Academic Press, 1993).

24. Landete-Castillejos, null, Garciá, null, Garde, null & Gallego, null. Milk intake and production curves and allosuckling in captive Iberian red deer, Cervus elaphus hispanicus. *Anim. Behav.* **60**, 679–687 (2000).

25. Landete-Castillejos, T., García, A., Gómez, J. Á. & Gallego, L. Lactation under food constraints in Iberian red deer Cervus elaphus hispanicus. *Wildl. Biol.* **9**, 131–139 (2003).

26. Arman, P., Kay, R. N. B., Goodall, E. D. & Sharman, G. a. M. THE COMPOSITION AND YIELD OF MILK FROM CAPTIVE RED DEER (CERVUS ELAPHUS L.). *Reproduction* **37**, 67–84 (1974).

27. Mueller, C. C. & Sadleir, R. M. F. S. Changes in the Nutrient Composition of Milk of Black-Tailed Deer during Lactation. *J. Mammal.* **58**, 421–423 (1977).

28. Arman, P. Milk from semi-domesticated ruminants. *World Rev. Nutr. Diet.* **33**, 198–227 (1979).

29. Carrión, D., García, A. J., Gaspar-López, E., Landete-Castillejos, T. & Gallego, L. Development of body condition in hinds of Iberian red deer during gestation and its effects on calf birth weight and milk production. *J. Exp. Zool. Part Ecol. Genet. Physiol.* **309**, 1–10 (2008).

30. Linzell, J. L. Milk yield, energy loss in milk, and mammary gland weight in different species. *Dairy Sci Abstr* **34**, 251–360 (1972).

31. de la Vara, J. A. *et al.* Some aspects of the ethanol stability of red deer milk (Cervus elaphus hispanicus): a comparison with other dairy species. *Int. Dairy J.* (2018) doi:10.1016/j.idairyj.2018.07.006.

32. Robbins, C. T., Oftedal, O. T. & O´Rourke, K. I. Lactation, early nutrition, and hand-rearing of wild ungulates, with special reference to deer. in *Biology and management of the Cervidae (C.M. Wemmer)* 429–442 (Smithsonian Institution Press, 1987).

33. Skibiel, A. L., Downing, L. M., Orr, T. J. & Hood, W. R. The evolution of the nutrient composition of mammalian milks. *J. Anim. Ecol.* **82**, 1254–1264 (2013).

34. Perrin, D. R. 709. The calorific value of milk of different species. *J. Dairy Res.* **25**, 215–220 (1958).

35. Wood, S. N. Fast stable restricted maximum likelihood and marginal likelihood estimation of semiparametric generalized linear models. *J. R. Stat. Soc. Ser. B Stat. Methodol.* **73**, 3–36 (2011).

36. Bates, D., Maechler, M., Bolker, B. & Walker, S. Fitting Linear Mixed-Effects Models Using lme4. *J. Stat. Softw.* **67**, 1–48 (2015).

37. R Core Team. *R: A Language and Environment for Statistical Computing, v. 3.4.1.* (R Foundation for Statistical Computing, 2017).

38. Kuznetsova, A., Brockhoff, B. & Christensen, R. H. B. *lmerTest: Tests in Linear Mixed Effects Models. R package version 2.0-29*. (2015).

39. Baayen, R. H., Davidson, D. J. & Bates, D. M. Mixed-effects modeling with crossed random effects for subjects and items. *J. Mem. Lang.* **59**, 390–412 (2008).

40. Nakagawa, S. & Schielzeth, H. A general and simple method for obtaining R^2 from generalized linear mixed-effects models. *Methods Ecol. Evol.* **4**, 133–142 (2013).

41. Murtaugh, P. A. In defense of P values. *Ecology* **95**, 611–617 (2014).

42. Pérez-Barbería, F. J., Carranza, J. & Sánchez-Prieto, C. Wear Fast, Die Young: More Worn Teeth and Shorter Lives in Iberian Compared to Scottish Red Deer. *Plos One* **10**, e0134788 (2015).

43. Carranza, J. *et al.* Social environment modulates investment in sex trait versus lifespan: red deer produce bigger antlers when facing more rivalry. *Sci. Rep.* **10**, 9234 (2020).

44. Anderson, A. E., Medin, D. E. & Bowden, D. C. Growth and morphometry of the carcass, selected bones, organs and glands of mule deer. *Wildl. Monogr.* **39**, 1–122 (1974).

45. Geist, V. *Deer of the World: their Evolution, Behavior & Ecology*. (Stackpole Books, 1998).

46. Ringberg, T. M., White, R. G., Holleman, D. F. & Luick, J. R. Body Growth and carcass composition of lean reindeer (_Rangifer tarandus tarandus L) from birth to sexual maturity. *Can. J. Zool.* **59**, 1040–1044 (1981).

47. Pinheiro, J., Bates, D., DebRoy, S., Sarkar, D. & Team, R. C. *nlme: Linear and nonlinear mixed effects models. R package version 3.1-131. Retrieved on 229 July 2017 from http://CRAN.R-project.org/package=nlme*. (2017).

48. Pérez-Barbería, F. J. *et al.* Heat stress reduces growth rate of red deer calf: Climate warming implications. *PLOS ONE* **15**, e0233809 (2020).
