# Supplementary material for: Effects of maternal age and offspring sex on milk yield, composition and calf growth of red deer (Cervus elaphus)
Source: Sci Rep. 2022 Aug 25;12:14506. doi: 10.1038/s41598-022-17978-3 (PMC9411626; doi:10.1038/s41598-022-17978-3)
Supplement: Supplementary file 2 — Supplementary Information 2. [file 41598_2022_17978_MOESM2_ESM.docx]

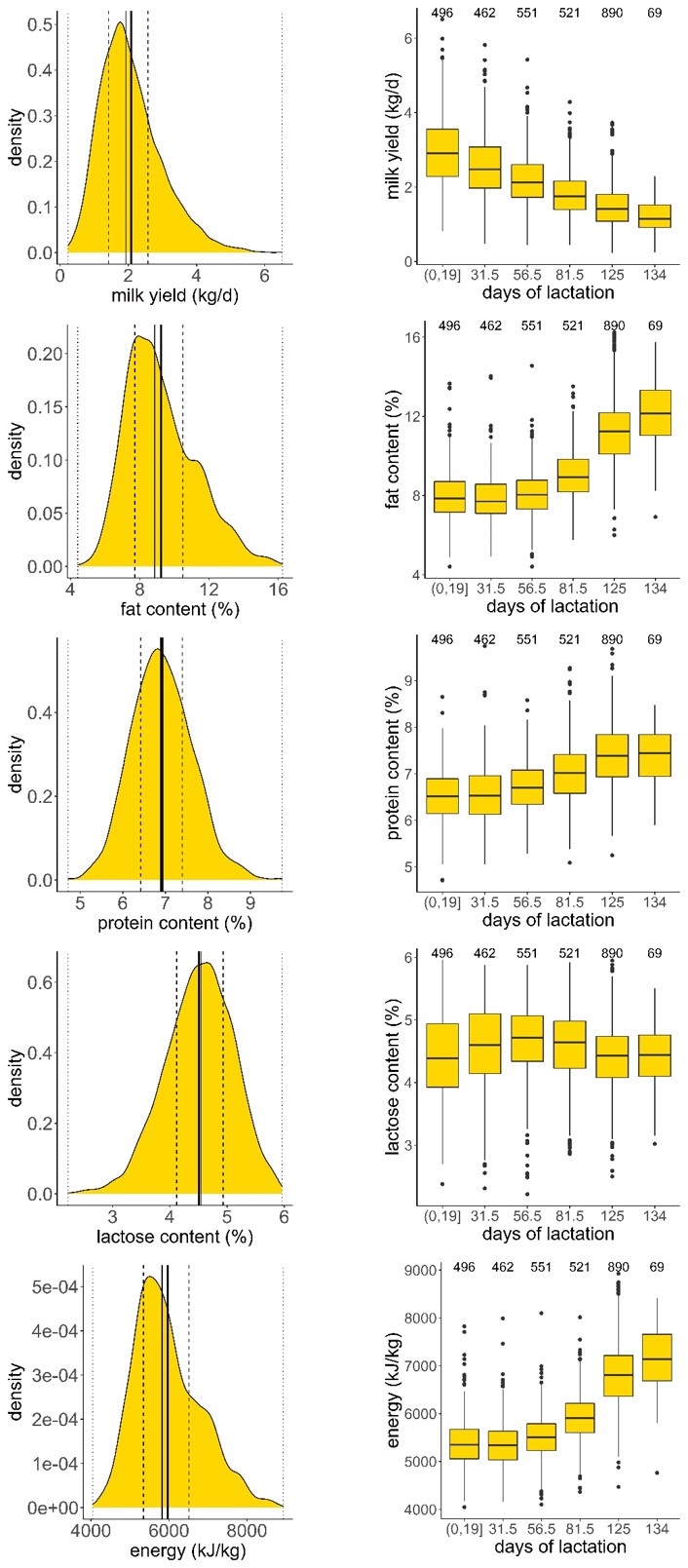


**ESM Figure 1.** Milk traits, yield (kg d^-1^), percentage of fat, protein, lactose, and density energy (kJ kg^-1^). Kernel density plots: dotted line = range (minimum, maximum); dashed line = 25 % and 75 % quantiles; solid thin line = 50 % quantile; solid thick line = mean. Box plots: horizontal middle line = 50 % quantile; box hinges = 25 % and 75 % quantiles; lower whisker = smallest observation greater than or equal to lower hinge minus 1.5 × inter-quartile range; upper whisker = largest observation less than or equal to upper hinge plus 1.5 × inter-quartile range. Black dots are outliers. X-axis labels are abbreviated break values of six day-periods across lactation (0 - 19], (19 - 31.5], (31.5 - 56.5], (56.5 - 81.5], (81.5 - 125], (125 - 134].

##
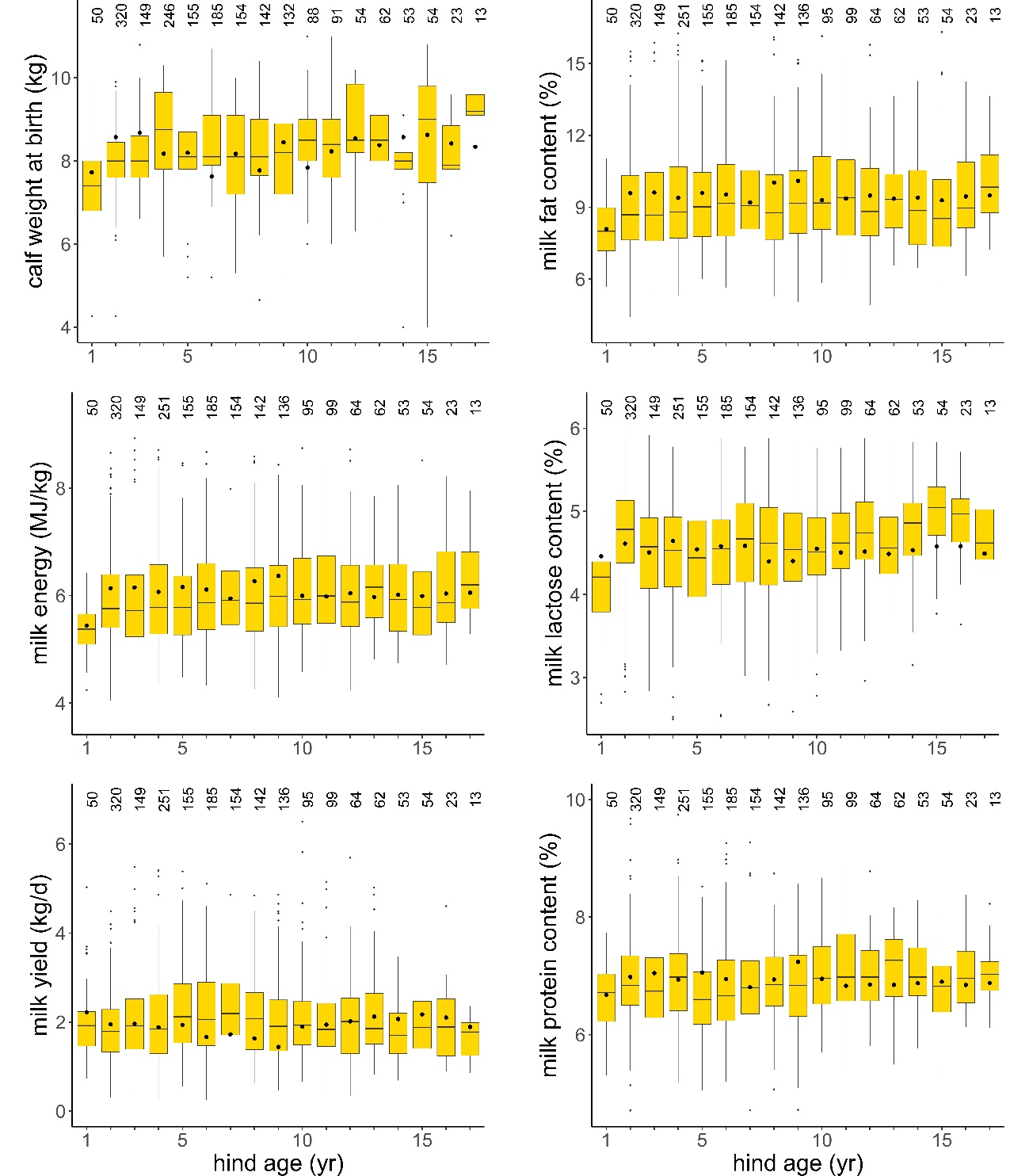


**ESM Figure 2.** Calf weight at birth and milk traits [yield (kg d^-1^), percentage of fat, protein, lactose, and density energy (kJ kg^-1^)] against hind age. Large dots are predictions of a linear mixed model that includes hind identity and year of lactation as the random effects. Sample size on top of the boxes. Box plots statistics as in ESM Figure 3.


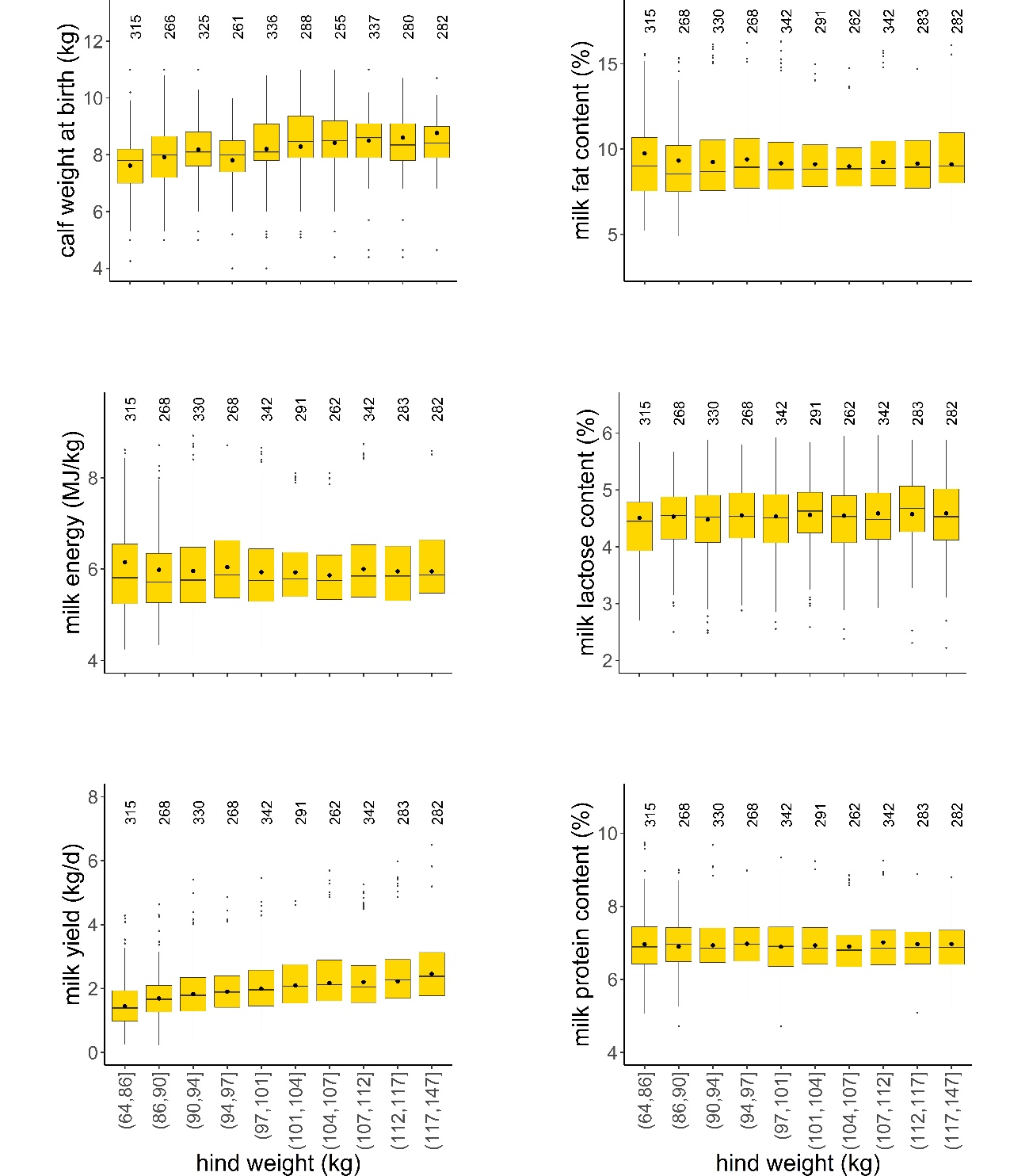


**ESM Figure 3.** Calf weight at birth and milk traits [yield (kg d^-1^), percentage of fat, protein, lactose, and density energy (kJ kg^-1^)] against hind body weight. Large dots are predictions of a mixed linear model that includes hind identity and year of lactation as the random effects. Sample size on top of the boxes. Box plots statistics as in ESM Figure 3.


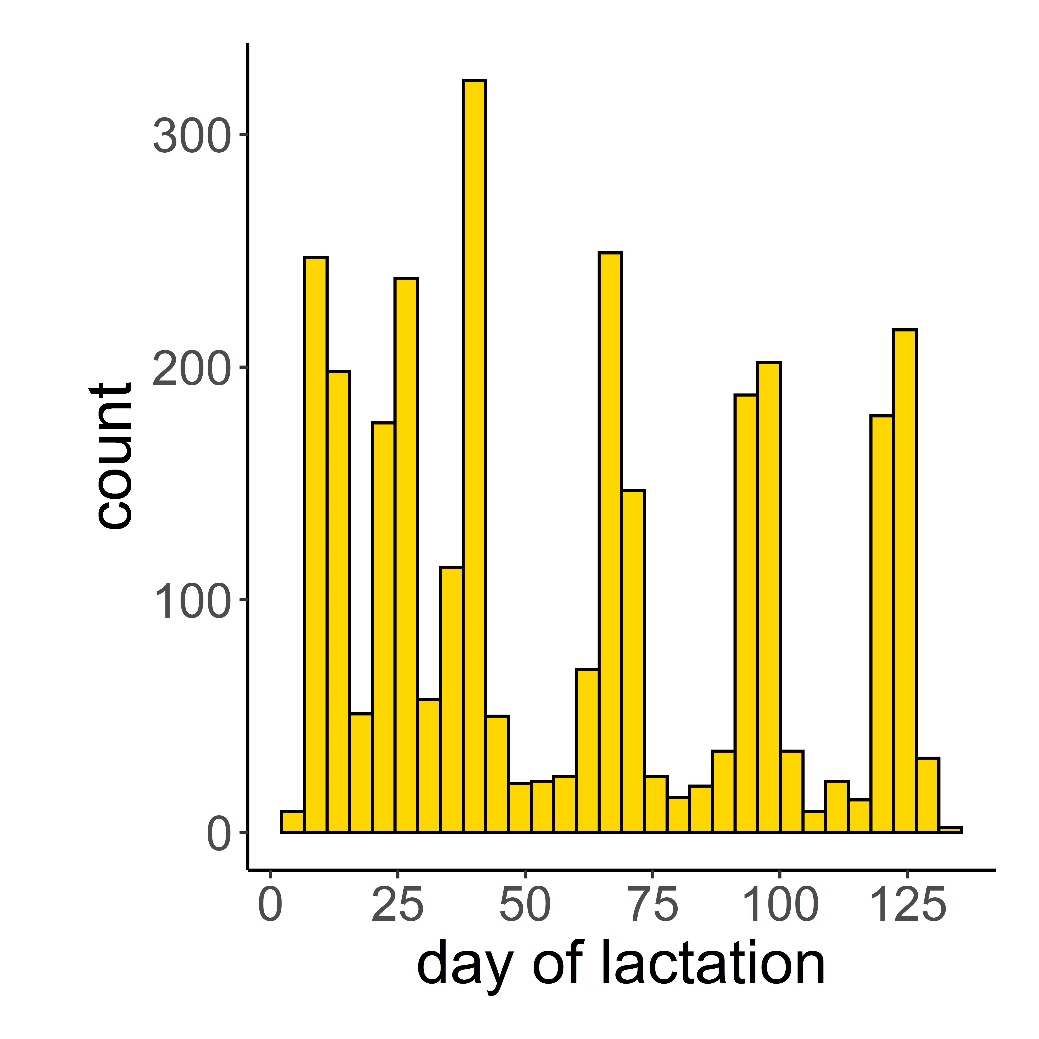


**ESM Figure 4.** Frequency distribution of milking events (days after parturition) along lactation for the study period.
