# Supplementary material for: Effects of maternal age and offspring sex on milk yield, composition and calf growth of red deer (Cervus elaphus)
Source: Sci Rep. 2022 Aug 25;12:14506. doi: 10.1038/s41598-022-17978-3 (PMC9411626; doi:10.1038/s41598-022-17978-3)
Supplement: Supplementary file 3 — Supplementary Information 3. [file 41598_2022_17978_MOESM3_ESM.docx]

**ESM Table 1.** Coefficients and statistics of an exponential asymptotic mixed linear model on calf weight (kg) controlling for hind weight (hind wt, kg), hind age (years), parity, parturition date (parturition) and calf sex (sex). Calf sex reference is female. See ESM Material 8 for details.

| **Random effects** | **sd** |  |  |  |  |
| --- | --- | --- | --- | --- | --- |
| Asym (intercept) | 13.367 |  |  |  |  |
| R0 (intercept) | 1.485 |  |  |  |  |
| Residual | 1.150 |  |  |  |  |
| Fixed effects | estimate | se | df | t-value | P |
| Asym (intercept) | 47.246 | 9.766 | 9628 | 4.84 | **<0.001** |
| Asym sex (male) | 12.269 | 13.745 | 9628 | 0.89 | 0.372 |
| Asym hind wt | 0.331 | 0.050 | 9628 | 6.66 | **<0.001** |
| Asym hind age | -1.728 | 0.656 | 9628 | -2.63 | **0.009** |
| Asym parity | 1.828 | 0.880 | 9628 | 2.08 | **0.038** |
| Asym parturition | -0.034 | 0.054 | 9628 | -0.64 | 0.520 |
| Asym hind wt x sex (male) | 0.312 | 0.071 | 9628 | 4.41 | **<0.001** |
| Asym hind age x sex (male) | -2.525 | 0.981 | 9628 | -2.57 | **0.010** |
| Asym parity x sex (male) | 2.200 | 1.281 | 9628 | 1.72 | 0.086 |
| Asym parturition x sex (male) | -0.133 | 0.075 | 9628 | -1.77 | 0.077 |
| R0 intercept | 7.080 | 1.103 | 9628 | 6.42 | **<0.001** |
| R0 sex (male) | 0.045 | 1.503 | 9628 | 0.03 | 0.976 |
| R0 hind wt | 0.019 | 0.007 | 9628 | 2.55 | **0.011** |
| R0 hind age | 0.142 | 0.068 | 9628 | 2.10 | **0.036** |
| R0 parity | -0.095 | 0.090 | 9628 | -1.06 | 0.290 |
| R0 parturition | -0.006 | 0.005 | 9628 | -1.15 | 0.251 |
| R0 hind wt x sex (male) | -0.003 | 0.010 | 9628 | -0.27 | 0.786 |
| R0 hind age x sex (male) | -0.102 | 0.095 | 9628 | -1.07 | 0.287 |
| R0 parity x sex (male) | 0.213 | 0.123 | 9628 | 1.73 | 0.084 |
| R0 parturition x sex (male) | 0.005 | 0.007 | 9628 | 0.69 | 0.490 |
| lrc intercept | -4.901 | 0.169 | 9628 | -29.03 | **<0.001** |
| lrc sex (male) | -0.091 | 0.214 | 9628 | -0.42 | 0.672 |
| lrc hind wt | -0.003 | 0.001 | 9628 | -3.09 | **0.002** |
| lrc hind age | 0.014 | 0.011 | 9628 | 1.28 | 0.199 |
| lrc parity | -0.008 | 0.014 | 9628 | -0.57 | 0.567 |
| lrc parturition | -0.001 | 0.001 | 9628 | -0.60 | 0.546 |
| lrc hind wt x sex (male) | -0.002 | 0.001 | 9628 | -1.84 | 0.066 |
| lrc hind age x sex (male) | 0.024 | 0.015 | 9628 | 1.67 | 0.096 |
| lrc parity x sex (male) | -0.025 | 0.018 | 9628 | -1.34 | 0.181 |
| lrc parturition x sex (male) | 0.001 | 0.001 | 9628 | 0.91 | 0.360 |
| body weight records = 10297  calves = 635 |  |  |  |  |  |

**ESM Table 2.** Coefficients and statistics of a polynomial mixed model of degree 3 on milk yield (kg/d) controlling for hind weight (hind wt, kg), hind age (years), parity, parturition date (parturition), calf sex (sex), calf weight (calf wt), day of lactation (lactation day), calf, hind and mother of hind identity (calf ID, hind ID, mother of hind ID) and year. Calf sex reference is male. CI, 95% confident interval of estimates; Marginal *R^2^*, variance accounted for by the fixed effects; Conditional *R^2^*, variance accounted for by random and fixed effects.

| *Predictors* | *Coefficients* | *CI* | *p* |
| --- | --- | --- | --- |
| Intercept | 2.15 | 2.04 – 2.27 | **<0.001** |
| Hind age [1] | -4.45 | -9.60 – 0.70 | 0.090 |
| Hind age [2] | 1.88 | -1.54 – 5.30 | 0.282 |
| Hind age [3] | -2.80 | -5.78 – 0.17 | 0.065 |
| Hind wt [1] | 12.77 | 10.14 – 15.41 | **<0.001** |
| Hind wt [2] | -0.19 | -1.95 – 1.58 | 0.836 |
| Hind wt [3] | 0.22 | -1.25 – 1.69 | 0.768 |
| Parity [1] | 1.17 | -3.42 – 5.77 | 0.617 |
| Parity [2] | -1.89 | -4.41 – 0.62 | 0.140 |
| Parity [3] | 1.87 | -0.19 – 3.93 | 0.075 |
| Parturition [1] | -3.52 | -5.43 – -1.61 | **<0.001** |
| Parturition [2] | -1.52 | -3.17 – 0.14 | 0.072 |
| Parturition [3] | 0.90 | -0.83 – 2.63 | 0.308 |
| Calf wt [1] | 7.13 | 1.89 – 12.37 | **0.008** |
| Calf wt [2] | -4.37 | -6.60 – -2.13 | **<0.001** |
| Calf wt [3] | 1.72 | 0.22 – 3.23 | **0.025** |
| Lactation day [1] | -30.76 | -35.98 – -25.54 | **<0.001** |
| Lactation day [2] | 10.36 | 7.89 – 12.82 | **<0.001** |
| Lactation day [3] | -5.20 | -6.89 – -3.51 | **<0.001** |
| Sex [male] | -0.04 | -0.13 – 0.04 | 0.338 |
| Hind age [1] x Sex [male] | -0.68 | -4.03 – 2.67 | 0.692 |
| Hind age [2] x Sex [male] | 0.43 | -3.10 – 3.97 | 0.810 |
| Hind age [3] x Sex [male] | 0.02 | -3.51 – 3.55 | 0.990 |
| Lactation day [1] x Sex [male] | 1.42 | -0.81 – 3.65 | 0.213 |
| Lactation day [2] x Sex [male] | 0.24 | -1.92 – 2.40 | 0.830 |
| Lactation day [3] x Sex [male] | 2.02 | -0.03 – 4.07 | 0.054 |
| Hind age [1] x Lactation day [1] | 8.40 | -36.07 – 52.87 | 0.711 |
| Hind age [2] x Lactation day [1] | 99.39 | 54.06 – 144.73 | **<0.001** |
| Hind age [3] x Lactation day [1] | -75.80 | -119.45 – -32.14 | **0.001** |
| Hind age [1] x Lactation day [2] | -36.12 | -79.16 – 6.91 | 0.100 |
| Hind age [2] x Lactation day [2] | -4.13 | -47.64 – 39.38 | 0.852 |
| Hind age [3] x Lactation day [2] | 35.84 | -7.41 – 79.10 | 0.104 |
| Hind age [1] x Lactation day [3] | 44.04 | 1.32 – 86.76 | **0.043** |
| Hind age [2] x Lactation day [3] | -46.65 | -89.70 – -3.61 | **0.034** |
| Hind age [3] x Lactation day [3] | -16.66 | -60.02 – 26.70 | 0.451 |
| **Random Effects** | | | |
| Residual variance | 0.26 | | |
| Calf ID | 0.04 | | |
| Hind ID | 0.04 | | |
| Mother of hind ID | 0.02 | | |
| Year | 0.02 | | |
| N hinds | 81 | | |
| N calves | 267 | | |
| N year | 18 | | |
| N mother of hind | 57 | | |
| Observations | 1715 | | |
| Marginal R^2^ / Conditional R^2^ | 0.552 / 0.694 | | |

**ESM Table 3.** Coefficients and statistics of a polynomial mixed model on milk density energy (MJ kg^-1^). Other acronyms as in SEM Table 2.

| *Predictors* | *Coefficients* | *CI* | *p* |
| --- | --- | --- | --- |
| Intercept | 6.04 | 5.92 – 6.16 | **<0.001** |
| Hind age [1] | -4.95 | -9.66 – -0.25 | **0.039** |
| Hind age [2] | 1.57 | -1.06 – 4.21 | 0.242 |
| Hind age [3] | -1.15 | -3.45 – 1.15 | 0.329 |
| Hind wt [1] | 0.02 | -2.28 – 2.32 | 0.989 |
| Hind wt [2] | 1.24 | -0.21 – 2.68 | 0.095 |
| Hind wt [3] | -0.81 | -2.01 – 0.38 | 0.184 |
| Parity [1] | 3.58 | -0.52 – 7.68 | 0.087 |
| Parity [2] | -2.12 | -4.02 – -0.21 | **0.029** |
| Parity [3] | 0.53 | -1.04 – 2.09 | 0.511 |
| Parturition [1] | 1.12 | -0.37 – 2.60 | 0.140 |
| Parturition [2] | -0.44 | -1.70 – 0.83 | 0.498 |
| Parturition [3] | -1.14 | -2.49 – 0.20 | 0.095 |
| Calf wt [1] | -2.98 | -7.17 – 1.22 | 0.165 |
| Calf wt [2] | -1.58 | -3.42 – 0.26 | 0.093 |
| Calf wt [3] | 1.57 | 0.31 – 2.84 | **0.015** |
| Lactation day [1] | 29.45 | 25.25 – 33.66 | **<0.001** |
| Lactation day [2] | 7.65 | 5.60 – 9.70 | **<0.001** |
| Lactation day [3] | -3.87 | -5.31 – -2.44 | **<0.001** |
| Sex [male] | -0.04 | -0.11 – 0.02 | 0.174 |
| Hind age [1] x Sex [male] | 0.59 | -1.94 – 3.13 | 0.647 |
| Hind age [2] x Sex [male] | -0.01 | -2.70 – 2.68 | 0.995 |
| Hind age [3] x Sex [male] | 1.58 | -1.13 – 4.29 | 0.254 |
| Lactation day [1] x Sex [male] | 0.37 | -1.53 – 2.26 | 0.705 |
| Lactation day [2] x Sex [male] | -2.09 | -3.93 – -0.26 | **0.025** |
| Lactation day [3] x Sex [male] | -0.38 | -2.12 – 1.37 | 0.673 |
| Hind age [1] x Lactation day [1] | -59.16 | -96.84 – -21.49 | **0.002** |
| Hind age [2] x Lactation day [1] | 49.73 | 11.42 – 88.03 | **0.011** |
| Hind age [3] x Lactation day [1] | 4.21 | -32.88 – 41.30 | 0.824 |
| Hind age [1] x Lactation day [2] | -86.17 | -122.81 – -49.54 | **<0.001** |
| Hind age [2] x Lactation day [2] | 46.11 | 9.08 – 83.14 | **0.015** |
| Hind age [3] x Lactation day [2] | 22.50 | -14.32 – 59.32 | 0.231 |
| Hind age [1] x Lactation day [3] | 0.45 | -35.90 – 36.80 | 0.981 |
| Hind age [2] x Lactation day [3] | 11.08 | -25.54 – 47.70 | 0.553 |
| Hind age [3] x Lactation day [3] | 11.56 | -25.31 – 48.42 | 0.539 |
| **Random Effects** | | | |
| Residual variance | 0.19 | | |
| Calf ID | 0.01 | | |
| Hind ID | 0.07 | | |
| Mother of hind ID | 0.00 | | |
| Year | 0.04 | | |
| N hinds | 81 | | |
| N calves | 267 | | |
| N year | 18 | | |
| N mother of hind | 57 | | |
| Observations | 1715 | | |
| Marginal R^2^ / Conditional R^2^ | 0.701 / 0.774 | | |

**ESM Table 4.** Coefficients and statistics of a polynomial mixed model on milk fat percentage. Other acronyms as in SEM Table 2.

| *Predictors* | *Coefficients* | *CI* | *p* |
| --- | --- | --- | --- |
| Intercept | 9.41 | 9.12 – 9.70 | **<0.001** |
| Hind age [1] | -12.63 | -24.17 – -1.09 | **0.032** |
| Hind age [2] | 2.87 | -3.78 – 9.51 | 0.398 |
| Hind age [3] | -2.49 | -8.29 – 3.31 | 0.400 |
| Hind wt [1] | -1.04 | -6.70 – 4.62 | 0.719 |
| Hind wt [2] | 3.38 | -0.21 – 6.98 | 0.065 |
| Hind wt [3] | -0.78 | -3.75 – 2.18 | 0.605 |
| Parity [1] | 8.34 | -1.72 – 18.41 | 0.104 |
| Parity [2] | -5.13 | -9.89 – -0.36 | **0.035** |
| Parity [3] | 1.37 | -2.55 – 5.28 | 0.494 |
| Parturition [1] | 1.08 | -2.62 – 4.79 | 0.566 |
| Parturition [2] | -0.61 | -3.76 – 2.55 | 0.707 |
| Parturition [3] | -2.61 | -5.96 – 0.74 | 0.126 |
| Calf wt [1] | -12.72 | -23.15 – -2.29 | **0.017** |
| Calf wt [2] | -3.23 | -7.79 – 1.33 | 0.165 |
| Calf wt [3] | 4.95 | 1.82 – 8.08 | **0.002** |
| Lactation day [1] | 74.25 | 63.81 – 84.69 | **<0.001** |
| Lactation day [2] | 19.99 | 14.91 – 25.07 | **<0.001** |
| Lactation day [3] | -11.03 | -14.58 – -7.47 | **<0.001** |
| Sex [male] | -0.12 | -0.28 – 0.04 | 0.143 |
| Hind age [1] x Sex [male] | 2.56 | -3.84 – 8.95 | 0.433 |
| Hind age [2] x Sex [male] | 0.08 | -6.75 – 6.90 | 0.982 |
| Hind age [3] x Sex [male] | 4.27 | -2.64 – 11.18 | 0.226 |
| Lactation day [1] x Sex [male] | 0.81 | -3.88 – 5.49 | 0.736 |
| Lactation day [2] x Sex [male] | -5.54 | -10.07 – -1.00 | **0.017** |
| Lactation day [3] x Sex [male] | -0.75 | -5.06 – 3.55 | 0.731 |
| Hind age [1] x Lactation day [1] | -190.56 | -332.42 – -48.71 | **0.008** |
| Hind age [2] x Lactation day [1] | 130.04 | -24.71 – 284.79 | 0.099 |
| Hind age [3] x Lactation day [1] | 14.35 | -145.76 – 174.45 | 0.860 |
| Hind age [1] x Lactation day [2] | -181.74 | -323.91 – -39.57 | **0.012** |
| Hind age [2] x Lactation day [2] | 20.33 | -136.00 – 176.65 | 0.799 |
| Hind age [3] x Lactation day [2] | -7.62 | -169.87 – 154.63 | 0.927 |
| Hind age [1] x Lactation day [3] | 16.02 | -122.16 – 154.20 | 0.820 |
| Hind age [2] x Lactation day [3] | 38.84 | -113.20 – 190.87 | 0.616 |
| Hind age [3] x Lactation day [3] | 27.38 | -133.55 – 188.31 | 0.739 |
| **Random Effects** | | | |
| Residual variance | 1.16 | | |
| Calf ID | 0.07 | | |
| Hind ID | 0.38 | | |
| Mother of hind ID | 0.00 | | |
| Year | 0.22 | | |
| N hinds | 81 | | |
| N calves | 267 | | |
| N year | 18 | | |
| N mother of hind | 57 | | |
| Observations | 1715 | | |
| Marginal R^2^ / Conditional R^2^ | 0.686 / 0.723 | | |

**ESM Table 5.** Coefficients and statistics of a polynomial mixed model on milk protein percentage. Other acronyms as in SEM Table 2.

| *Predictors* | *Coefficients* | *CI* | *p* |
| --- | --- | --- | --- |
| Intercept | 6.92 | 6.75 – 7.08 | **<0.001** |
| Hind age [1] | -1.31 | -6.40 – 3.78 | 0.614 |
| Hind age [2] | 1.92 | -0.73 – 4.56 | 0.156 |
| Hind age [3] | -0.71 | -3.00 – 1.59 | 0.547 |
| Hind wt [1] | 0.80 | -1.61 – 3.21 | 0.515 |
| Hind wt [2] | -0.32 | -1.78 – 1.13 | 0.662 |
| Hind wt [3] | -1.34 | -2.53 – -0.15 | **0.027** |
| Parity [1] | 0.81 | -3.58 – 5.21 | 0.717 |
| Parity [2] | -0.82 | -2.73 – 1.08 | 0.396 |
| Parity [3] | 0.23 | -1.33 – 1.80 | 0.772 |
| Parturition [1] | 2.41 | 0.91 – 3.91 | **0.002** |
| Parturition [2] | -1.02 | -2.29 – 0.25 | 0.115 |
| Parturition [3] | -0.31 | -1.67 – 1.05 | 0.655 |
| Calf wt [1] | 6.71 | 2.55 – 10.87 | **0.002** |
| Calf wt [2] | 1.00 | -0.81 – 2.81 | 0.279 |
| Calf wt [3] | -1.89 | -3.13 – -0.64 | **0.003** |
| Lactation day [1] | 8.74 | 4.57 – 12.90 | **<0.001** |
| Lactation day [2] | -0.41 | -2.42 – 1.61 | 0.692 |
| Lactation day [3] | -0.01 | -1.41 – 1.40 | 0.993 |
| Sex [male] | 0.01 | -0.06 – 0.07 | 0.870 |
| Hind age [1] x Sex [male] | -0.56 | -3.10 – 1.98 | 0.666 |
| Hind age [2] x Sex [male] | 0.19 | -2.50 – 2.88 | 0.887 |
| Hind age [3] x Sex [male] | -0.18 | -2.88 – 2.53 | 0.899 |
| Lactation day [1] x Sex [male] | -1.45 | -3.30 – 0.41 | 0.126 |
| Lactation day [2] x Sex [male] | 0.25 | -1.54 – 2.04 | 0.784 |
| Lactation day [3] x Sex [male] | -0.64 | -2.35 – 1.06 | 0.461 |
| Hind age [1] x Lactation day [1] | 35.43 | -1.41 – 72.28 | 0.059 |
| Hind age [2] x Lactation day [1] | -33.99 | -71.46 – 3.48 | 0.075 |
| Hind age [3] x Lactation day [1] | 24.25 | -12.00 – 60.51 | 0.190 |
| Hind age [1] x Lactation day [2] | -36.17 | -71.96 – -0.38 | **0.048** |
| Hind age [2] x Lactation day [2] | -7.50 | -43.68 – 28.68 | 0.684 |
| Hind age [3] x Lactation day [2] | 4.41 | -31.57 – 40.39 | 0.810 |
| Hind age [1] x Lactation day [3] | 50.47 | 14.96 – 85.98 | **0.005** |
| Hind age [2] x Lactation day [3] | -29.99 | -65.76 – 5.77 | 0.100 |
| Hind age [3] x Lactation day [3] | 21.94 | -14.08 – 57.95 | 0.232 |
| **Random Effects** | | | |
| Residual variance | 0.18 | | |
| Calf ID | 0.01 | | |
| Hind ID | 0.09 | | |
| Mother of hind ID | 0.00 | | |
| Year | 0.09 | | |
| N hinds | 81 | | |
| N calves | 267 | | |
| N year | 18 | | |
| N mother of hind | 57 | | |
| Observations | 1715 | | |
| Marginal R^2^ / Conditional R^2^ | 0.261 / 0.641 | | |

**ESM Table 6.** Coefficients and statistics of a polynomial mixed model on milk lactose percentage. Other acronyms as in SEM Table 2.

|  | **lact** | | |
| --- | --- | --- | --- |
| *Predictors* | *Estimates* | *CI* | *p* |
| Intercept | 4.54 | 4.36 – 4.72 | **<0.001** |
| Hind age [1] | 1.16 | -2.15 – 4.46 | 0.493 |
| Hind age [2] | -0.90 | -3.05 – 1.26 | 0.414 |
| Hind age [3] | 0.14 | -1.78 – 2.06 | 0.886 |
| Hind wt [1] | 0.56 | -1.21 – 2.33 | 0.536 |
| Hind wt [2] | 0.13 | -1.06 – 1.33 | 0.826 |
| Hind wt [3] | -0.53 | -1.55 – 0.48 | 0.304 |
| Parity [1] | 0.62 | -2.33 – 3.56 | 0.681 |
| Parity [2] | 0.37 | -1.21 – 1.94 | 0.648 |
| Parity [3] | -0.47 | -1.77 – 0.82 | 0.475 |
| Parturition [1] | 0.60 | -0.60 – 1.80 | 0.326 |
| Parturition [2] | 0.44 | -0.59 – 1.47 | 0.400 |
| Parturition [3] | -0.32 | -1.40 – 0.76 | 0.560 |
| Calf wt [1] | 0.56 | -3.05 – 4.17 | 0.760 |
| Calf wt [2] | -2.88 | -4.57 – -1.20 | **0.001** |
| Calf wt [3] | 0.89 | -0.32 – 2.09 | 0.149 |
| Lactation day [1] | -5.02 | -8.67 – -1.36 | **0.007** |
| Lactation day [2] | -0.29 | -2.20 – 1.61 | 0.765 |
| Lactation day [3] | 1.93 | 0.56 – 3.31 | **0.006** |
| Sex [male] | 0.01 | -0.04 – 0.07 | 0.578 |
| Hind age [1] x Sex [male] | -1.60 | -3.65 – 0.45 | 0.125 |
| Hind age [2] x Sex [male] | -0.03 | -2.23 – 2.18 | 0.982 |
| Hind age [3] x Sex [male] | -0.14 | -2.42 – 2.14 | 0.906 |
| Lactation day [1] x Sex [male] | 2.37 | 0.55 – 4.18 | **0.011** |
| Lactation day [2] x Sex [male] | 0.10 | -1.67 – 1.86 | 0.915 |
| Lactation day [3] x Sex [male] | 0.49 | -1.19 – 2.17 | 0.567 |
| Hind age [1] x Lactation day [1] | 24.31 | -30.87 – 79.49 | 0.388 |
| Hind age [2] x Lactation day [1] | 28.31 | -31.85 – 88.48 | 0.356 |
| Hind age [3] x Lactation day [1] | 35.09 | -27.23 – 97.41 | 0.270 |
| Hind age [1] x Lactation day [2] | 49.83 | -5.53 – 105.18 | 0.078 |
| Hind age [2] x Lactation day [2] | 56.55 | -4.35 – 117.45 | 0.069 |
| Hind age [3] x Lactation day [2] | 25.01 | -38.24 – 88.26 | 0.438 |
| Hind age [1] x Lactation day [3] | -2.78 | -56.69 – 51.14 | 0.920 |
| Hind age [2] x Lactation day [3] | -7.43 | -66.75 – 51.90 | 0.806 |
| Hind age [3] x Lactation day [3] | -17.87 | -80.60 – 44.86 | 0.576 |
| Hind age [1] x Lactation day [1] x Sex [male] | -35.96 | -109.02 – 37.09 | 0.334 |
| Hind age [2] x Lactation day [1] x Sex [male] | -80.52 | -156.67 – -4.37 | **0.038** |
| Hind age [3] x Lactation day [1] x Sex [male] | -16.42 | -93.72 – 60.88 | 0.677 |
| Hind age [1] x Lactation day [2] x Sex [male] | -13.59 | -86.82 – 59.64 | 0.716 |
| Hind age [2] x Lactation day [2] x Sex [male] | -35.49 | -111.68 – 40.71 | 0.361 |
| Hind age [3] x Lactation day [2] x Sex [male] | -37.14 | -115.29 – 41.01 | 0.351 |
| Hind age [1] x Lactation day [3] x Sex [male] | -20.04 | -92.44 – 52.36 | 0.587 |
| Hind age [2] x Lactation day [3] x Sex [male] | -1.53 | -76.58 – 73.52 | 0.968 |
| Hind age [3] x Lactation day [3] x Sex [male] | 5.57 | -72.42 – 83.56 | 0.889 |
| **Random Effects** | | | |
| Residual variance | 0.18 | | |
| Calf ID | 0.00 | | |
| Hind ID | 0.02 | | |
| Mother of hind ID | 0.00 | | |
| Year | 0.14 | | |
| N hinds | 81 | | |
| N calves | 267 | | |
| N year | 18 | | |
| N mother of hind | 57 | | |
| Observations | 1715 | | |
| Marginal R^2^ / Conditional R^2^ | 0.056 / 0.505 | | |

**ESM Table 7.** Number of lactating red deer hinds per cohort.

| **Cohort** | **1996** | **1998** | **1999** | **2000** | **2001** | **2002** | **2003** | **2004** | **2005** | **2006** | **2007** | **2008** | **2009** | **2010** | **2011** | **2012** | **2013** | **2014** | **2016** | **2017** |
| --- | --- | --- | --- | --- | --- | --- | --- | --- | --- | --- | --- | --- | --- | --- | --- | --- | --- | --- | --- | --- |
| No. hinds | 5 | 10 | 5 | 12 | 7 | 17 | 6 | 21 | 22 | 6 | 9 | 1 | 1 | 8 | 1 | 2 | 3 | 7 | 11 | 2 |

**ESM Table 8.** Frequency distribution of the number of years within red deer hind in which milk yield, milk composition and calf growth were monitored between 1998 and 2020.

| **No. years** |  | **1** | **2** | **3** | **4** | **5** | **6** | **7** | **8** | **9** | **10** | **11** | **12** | **13** | **14** |
| --- | --- | --- | --- | --- | --- | --- | --- | --- | --- | --- | --- | --- | --- | --- | --- |
| No. hinds | Calf growth | 38 | 31 | 23 | 11 | 9 | 11 | 6 | 9 | 7 | 2 | 2 | 4 | 2 | 1 |
|  | Milk traits | 28 | 9 | 8 | 11 | 7 | 9 | 3 | 1 | 1 | 1 | 1 | 2 | - | - |
